# Cost-effectiveness of tenofovir gel in urban South Africa: model projections of HIV impact and threshold product prices

**DOI:** 10.1186/1471-2334-14-14

**Published:** 2014-01-09

**Authors:** Fern Terris-Prestholt, Anna M Foss, Andrew P Cox, Lori Heise, Gesine Meyer-Rath, Sinead Delany-Moretlwe, Thomas Mertenskoetter, Helen Rees, Peter Vickerman, Charlotte H Watts

**Affiliations:** 1 London School of Hygiene & Tropical Medicine, London, UK; 2 Health Economics and Epidemiology Research Office, Department of Medicine, Faculty of Health Sciences, University of the Witwatersrand, Johannesburg, South Africa; 3 Center for Global Health and Development, Boston University, Boston, USA; 4 Wits Reproductive Health and HIV Research Institute, Johannesburg, South Africa; 5 International Partnership for Microbicides, Silver Spring, MD, USA

**Keywords:** HIV, Modelling, Cost-effectiveness, Microbicides, South Africa, Introduction of new technologies, Economic analysis, Tenofovir, ARV-based prevention, Pre-exposure prophylaxis (PreP)

## Abstract

**Background:**

There is urgent need for effective HIV prevention methods that women can initiate. The CAPRISA 004 trial showed that a tenofovir-based vaginal microbicide had significant impact on HIV incidence among women. This study uses the trial findings to estimate the population-level impact of the gel on HIV and HSV-2 transmission, and price thresholds at which widespread product introduction would be as cost-effective as male circumcision in urban South Africa.

**Methods:**

The estimated ‘per sex-act’ HIV and HSV-2 efficacies were imputed from CAPRISA 004. A dynamic HIV/STI transmission model, parameterised and fitted to Gauteng (HIV prevalence of 16.9% in 2008), South Africa, was used to estimate the impact of gel use over 15 years. Uptake was assumed to increase linearly to 30% over 10 years, with gel use in 72% of sex-acts. Full economic programme and averted HIV treatment costs were modelled. Cost per DALY averted is estimated and a microbicide price that equalises its cost-effectiveness to that of male circumcision is estimated.

**Results:**

Using plausible assumptions about product introduction, we predict that tenofovir gel use could lead to a 12.5% and 4.9% reduction in HIV and HSV-2 incidence respectively, by year 15. Microbicide introduction is predicted to be highly cost-effective (under $300 per DALY averted), though the dose price would need to be just $0.12 to be equally cost-effective as male circumcision. A single dose or highly effective (83% HIV efficacy per sex-act) regimen would allow for more realistic threshold prices ($0.25 and $0.33 per dose, respectively).

**Conclusions:**

These findings show that an effective coitally-dependent microbicide could reduce HIV incidence by 12.5% in this setting, if current condom use is maintained. For microbicides to be in the range of the most cost-effective HIV prevention interventions, product costs will need to decrease substantially.

## Background

There is an urgent need for effective HIV prevention methods that can be initiated by women. After a decade of little progress in HIV prevention interventions, recent trials have shown that antiretrovirals can be used to prevent HIV incidence and infectivity [1-4]. In 2010, the CAPRISA 004 trial in Kwazulu Natal, South Africa, showed that the incidence of HIV infection among women randomised to using a topical gel containing 1% of the antiretroviral tenofovir before and after sex, was reduced by 39% (95% CI 6-60%). In addition, women’s risk of acquiring herpes simplex virus type-2 (HSV-2) was reduced by 51% (95% CI 22-70%) [5,6]. This finding was groundbreaking, being the first clinical trial to show a statistically significant impact of a microbicide product on HIV incidence, and providing a proof of concept that a topically applied antiretroviral microbicide can reduce women’s risk of HIV acquisition [7]. The FACTS 001 trial is now seeking to confirm the CAPRISA results, and assess the generalizability of the findings across different settings [8]. Plans are underway to prepare for the possible introduction and manufacture of tenofovir gel. Once this trial is completed, its data will be combined with the CAPRISA 004 results and presented together for regulatory approval. This study aims to inform this process and the field of HIV more broadly.

Evidence from FACTS 001 and this cost-effectiveness analysis will be evaluated in the context of VOICE trial results that showed that daily administration of the same product and oral tenofovir did not achieve a significant reduction in HIV incidence due to adherence issues [9]. It appears that compliance to a daily regimen of gels or pills is not a viable option for African women in particular for younger women. However, given the positive findings from CAPRISA, there is still hope that a coitally-specific product may provide a feasible strategy for women who are unable to protect themselves with the current range of options available.

In parallel to clinical research, it is important to explore the potential population-level impact that tenofovir gel introduction could have on HIV incidence in different epidemic settings, and identify key factors that may influence the impact and cost-effectiveness of future product introduction. Towards this end, this paper uses modelling and the CAPRISA trial results to estimate the gel’s likely level of protection against HIV and HSV-2 during each sex-act (here termed the ‘imputed efficacy’), and the potential population-level impact on HIV and HSV-2 of gradual product introduction in an urban setting with high HIV prevalence and incidence in South Africa. Using South Africa specific unit cost data, the full cost of introducing microbicides into the public health sector is modelled first. Secondly, threshold product prices are identified at which point the costs per HIV infection averted are equal to that of voluntary medical male circumcision (VMMC) (~$1000 per infection averted) [10], an intervention widely accepted as cost-effective and efficient. A range of scenarios are used to explore the effects of various assumptions on this threshold.

## Methods

### Setting

For the modelling analysis, we considered product introduction in Gauteng Province, a highly urbanised province of South Africa with a population of reproductive age (aged 15 – 45 years) of 5.7 million. HIV prevalence among those aged 15–49 was 16.9% in 2008 [11], with a higher prevalence among females (21.1%) than males (11.2%) documented in 2005 [12]. Commercial sex work is an important driver of the HIV epidemic in Gauteng [13], with behavioural surveys suggesting that 6.4% of female family-planning clinic attendees had transactional sex in the last four weeks [14].

### Microbicide introduction scenarios modelled

A review of evidence and discussions with key stakeholders in South Africa and internationally were used to inform the microbicide introduction strategies considered in this paper [15]. As described in more detail below, we considered the provision of a microbicide through the public health system, assuming that mass media campaigns are used to increase awareness of the product availability; health providers are trained to provide the product, and they then routinely provide HIV testing, and counsel HIV negative clients about product use and the importance of adherence and repeated HIV testing [15].

As in the CAPRISA 004 trial, we consider a coitally-dependent gel, with two doses used per sex-act. We assumed that the mean consistency of gel use is the same as in the CAPRISA 004 trial (two doses used in 72% of sex-acts)^a^, irrespective of the type of partnership. In addition, a more pessimistic scenario of 50% gel use was considered.

In the CAPRISA trial, reported condom use increased from 79% in the first six months to 84% in the last six months (P < 0.001) [5].^b^ In this analysis we considered two potential scenarios. Firstly, we assumed that condom use is unaffected by gel use and HIV testing (and remains at the levels detailed in Additional file 1: Table S2 of the appendix). Secondly, we consider more pessimistic assumptions that there is a 5% or 10% absolute reduction in condom use among microbicide users.

In this analysis, we consider the impact on HIV and HSV-2 incidence of population-level gel distribution over 15 years, starting in January 2013. We assume that, following regulatory approval, tenofovir is made available to HIV-negative sexually active women attending public health facilities, alongside regular HIV testing.

Based on a review of the evidence on the rate of uptake of condoms and other new health technologies [16], we assume that for the first 10 years the proportion of HIV-negative women using the gel increases linearly, plateauing at 30%, half the rate at which urban sexually active women are currently accessing modern contraceptives in Gauteng [17], for the remaining five years. A more optimistic scenario of 60% uptake after 10 years was also explored.

### Imputed efficacy calculations

The CAPRISA 004 trial results represent an estimate of the average protection achieved across all users in the active arm of the trial, including those who used the gel consistently or intermittently, and those who did not use it at all [18]. As a result, for the modelling we need to impute the level of protection provided by the product against HIV and HSV-2 in each sex-act between a susceptible woman and an infected partner (that is, how much it reduces the probability of transmission during each act of intercourse). This was estimated by dividing the trial effectiveness by the percentage of all reported sex-acts in which gel was used [5,6,18,19]. Details of this calculation and the assumptions made are provided in the online Additional file 1. The imputed efficacy estimates were then used in the dynamic impact model. We also investigated the contribution of the gel’s HSV-2 efficacy on HIV transmission and vice versa.

### Description of the model structure

The HIV and HSV-2 impact projections were obtained using a population-level deterministic compartmental HIV/STI transmission model, adapted to enable the impact of the gradual introduction of a microbicide to be explored [20]. The mathematics underlying the dynamic transmission model are described in Vickerman et al. [21], and in the online Additional file. In brief, the model divides the heterosexual population into subgroups, with stratifications by sex, level of sexual activity and HIV/STI infection status. Using data on the probabilities of disease transmission and progression from the epidemiological literature, established mathematical techniques are used to estimate how HIV spreads between the different subgroups over time [22,23]. The transmission of HSV-2, syphilis and gonorrhoea/chlamydia are simulated, including the facilitating effect of these STIs on HIV transmission, and the effect of HIV on increasing the rate of HSV-2 symptomatic recurrences and HSV-2 infectivity (during both asymptomatic and symptomatic stages of infection) [24]. The model also incorporates the higher HIV infectivity associated with primary and late-stage HIV infection (in the absence of HIV treatment), and the effect of ongoing STI treatment and antiretroviral therapy (ART) in the population on levels of HIV infectivity [25], of which more detail can be found in the online supplement.

### Fitting the model to the setting

**Figure 1:**
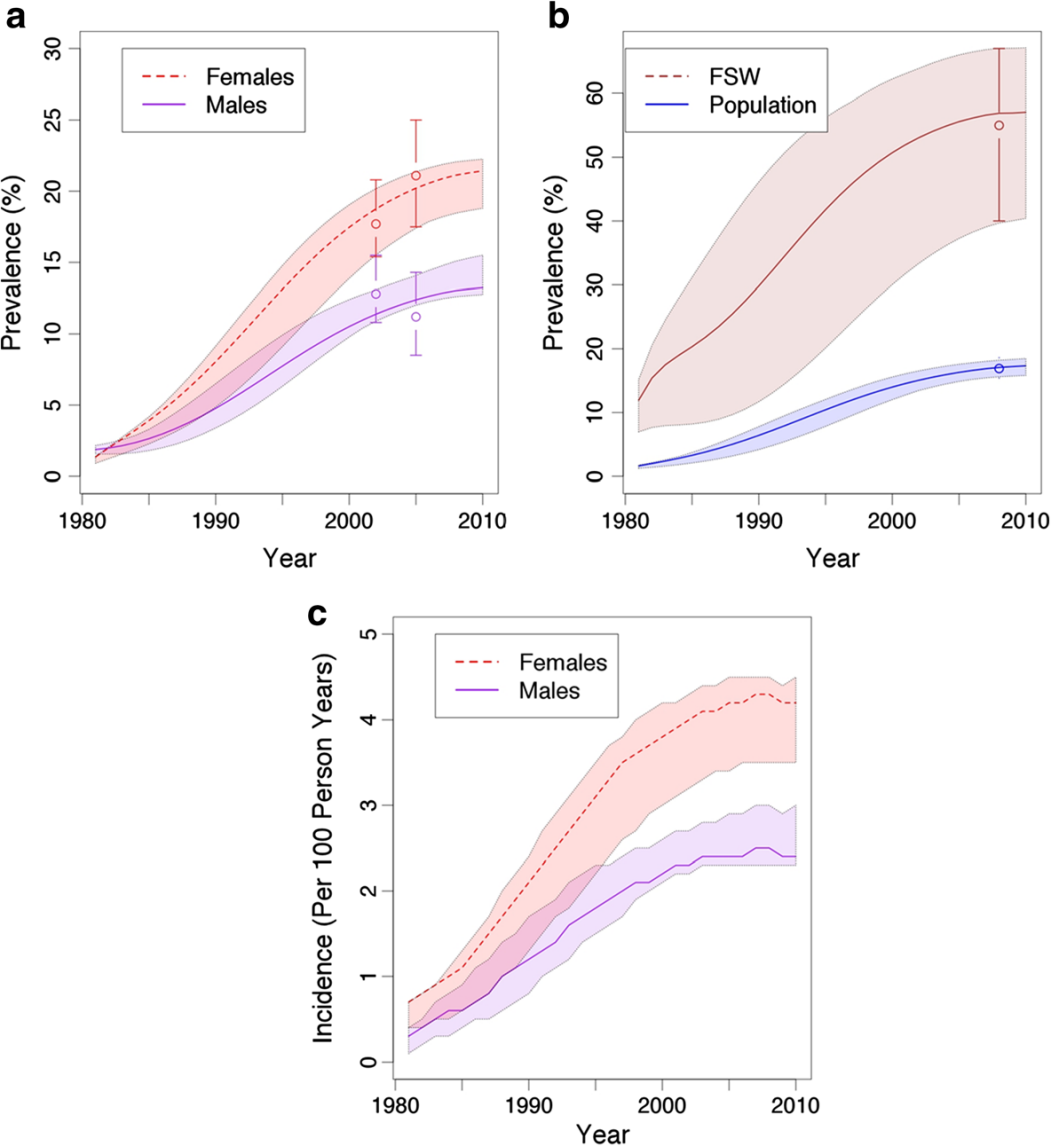
Model-projected HIV prevalence and incidence trends over time and data used to fit model*. a: HIV prevalence trends among general population males and females in 2002 and 2005. **b**: HIV prevalence trends among the general population and among female sex workers in 2008. **c**: HIV incidence trends among general population males and females. *The points with error bars for males and females in 2002 and 2005 represent the mean and 95% confidence intervals derived from data [12,26]. The dotted line represents the model-projected female HIV prevalence while the solid line represents the model-projected male HIV prevalence. The shaded areas represent the range of prevalence estimates spanned by all model fits.

The model was parameterised and fitted using behavioural and epidemiological data from Gauteng, and complementary data from South Africa (Figures 1a, b, c and online Additional file 1). The approach used incorporates the uncertainty inherent in key model parameters by running the model over a large number (almost 4 billion) of permutations of model inputs. These are obtained by randomly sampling from the uncertainty ranges for each model input parameter (Additional file 1: Tables S2-S4) [21]. As is standard in HIV modelling, the model is seeded with a few HIV infected individuals (0.5% in the general population and 5% among female sex workers) in 1980 and run until 2028 (to model 15 years from 2013). A ‘fit’ is defined as a model simulation that lies within the 95% confidence intervals (CIs) of setting-specific HIV and STI prevalence data. The model was fitted to HIV data from three national surveys, using disaggregated male and female prevalence values when available (for 2002, 2005) and a combined prevalence for 2008 (five HIV prevalence data points in total for the general population) [11,12,26]; and also to HIV prevalence data among female sex workers (FSWs) (1997) and their clients (2000). Additionally, the model was fitted to syphilis and gonorrhoea/chlamydia prevalence data from FSWs (1997) [13,27]. HSV-2 data from the CAPRISA trial [5], adjusting for HIV status [11], was used as a proxy for the general population HSV-2 prevalence. Each combination of input values that fit to the observed prevalence data is used in the final impact projections, with the best-fit identified through least chi-squared error.

### Methods used for the economic evaluation of gel introduction

Full programme costs are offset by the HIV care and treatment costs saved resulting from averting HIV infections to estimate incremental costs. We present the incremental cost-effectiveness ratios (ICER) in terms of dollar per disability adjusted life year (DALY) averted. Interventions with ICERs of less than per capita GDP ($7160 for South Africa) per averted DALY are considered highly cost-effective and worth introducing [28], but require either additional resources or re-allocation of resources. We also identify the threshold microbicide prices where the cost per HIV infection averted is equal to the cost per HIV infection averted of VMMC in South Africa to better understand under which conditions microbicide introduction would be as cost-effective as VMMC.

The economic costs of a comprehensive microbicide distribution programme were estimated from the provider’s perspective using an ingredients-based approach and unit cost from the South African public sector. This includes HIV testing [29], facility based provider training [30] and health facility costs, including staff, for the initiation visit and subsequent adherence counselling and gel distribution visits [31-33], 10% product wastage [34], and periodic mass media campaigns [35]. This analysis assumes general population HIV testing is in place and the initial screening costs are not included.

All costs are presented in 2012 US dollars ($). All future costs and effects are discounted to present values, using a 3% discount rate. The current price of a single dose of tenofovir gel as produced for the FACTS 001 trial is $1.60 [36]. The predicted price of a packaged and labelled gel at low production scale (10 million doses) is $0.56, while this could be as low as $0.17 if distributed at large scale with a tube of gel and fillable paper applicators [36].

For the 52% of those in need of ART who are receiving ART [37], 2012 annual treatment costs are used ($562 for first line ART and $1094 for second line) [38], with an lifetime cost of $14,310 assuming a near normal life expectancy due to ART [39]. For those without ART access, discounted lifetime HIV care costs of $3,080 are included [33]. This gives a discounted weighted average HIV care and ART costs averted of $8,920 per HIV infection averted. Though HSV-2 treatment with acyclovir is first line therapy for genital ulcer disease, these averted costs have not been included which will render the analysis on the conservative side.

An economic evaluation in terms of cost per DALY averted is estimated at a range of product prices with the central price being $0.25 (including $1.60, $0.56, and $0.17). Standard DALY estimates are used [40], with HIV disability weights drawn from the 2010 Global Burden of Disease study as presented in Ortblad [41]. Details of the economic evaluation methods are presented in the Additional file 1: Table S5. To approximate the price thresholds where microbicide introduction is roughly as cost effective as VMMC scale up ($1087 and $1096 per infection averted to scale up to 60% and 80%, respectively, from the current rate of 45%). To err on the conservative side, we use a $1,000 per infection averted threshold. Threshold prices are estimated under a range of assumptions (number of annual HIV tests and clinic visits (two to six times per year for both) and dosing (one to two doses per sex-act), and epidemiological scenarios). A sensitivity analysis is undertaken to understand the possible impact on the threshold price of: the prices of ART (-25%, -50%) and all other inputs (+/- 25%), discount rate (0%, 6%^c^), and treatment access rates (80%).

## Results

### Imputed efficacy and HIV incidence projections

**Figure 2:**
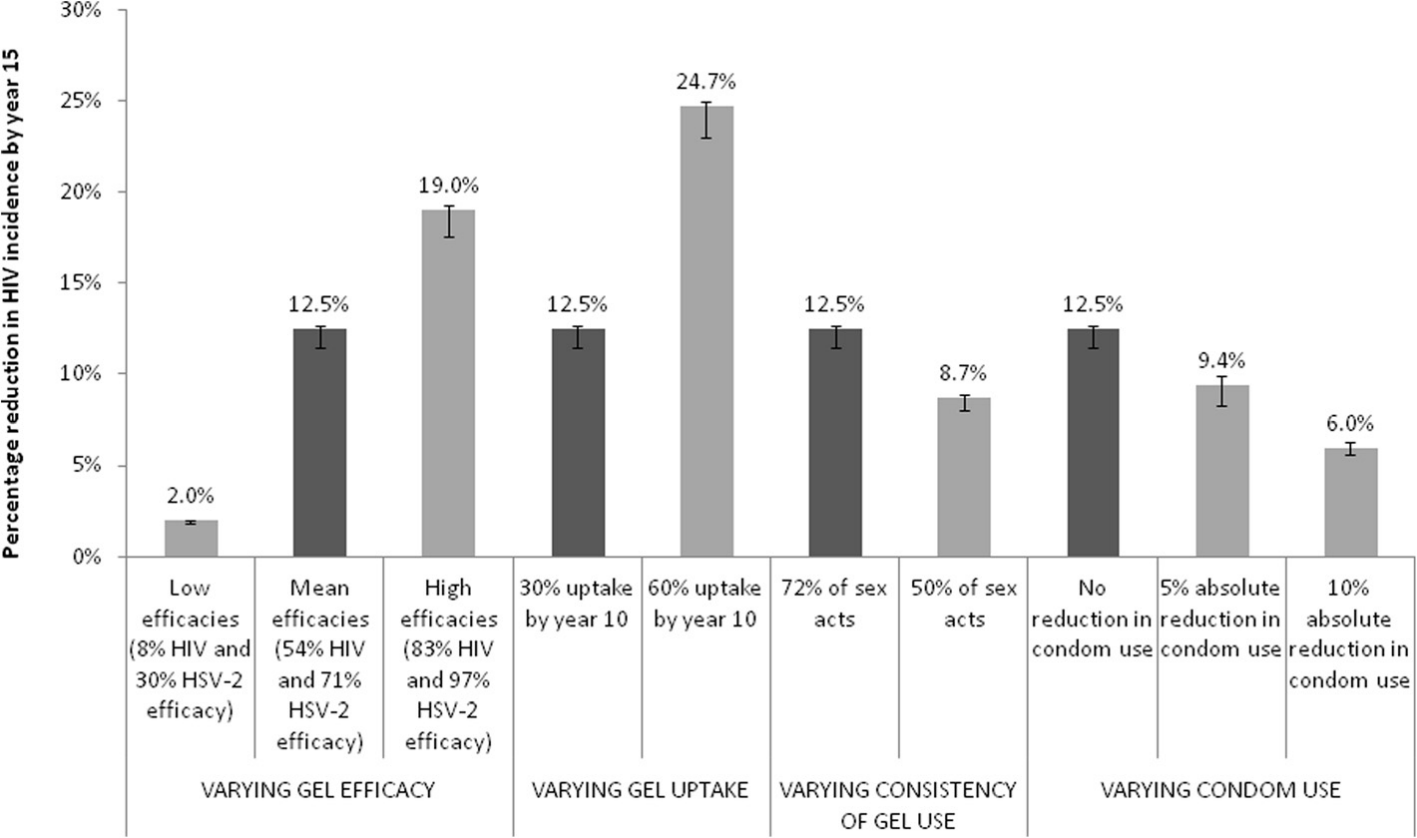
Projected population-level HIV impact in Gauteng for different assumptions about gel efficacy, uptake and use, and its influence on condom use**. **Main shaded bars show best-fit model projections and error bars indicate range spanned by 95% of all model fits (2.5% to 97.5% percentile range or 95% credibility interval), with the dark bars illustrating the main scenario (54% HIV efficacy and 71% HSV-2 efficacy, gel uptake reaching 30% by year 10, gel used in 72% of sex-acts, and no reduction in condom use).

The imputed efficacy calculations suggest that tenofovir gel reduced the per sex-act probability of HIV acquisition by 54% (95% CrI 8-83%) and the per sex-act probability of HSV-2 acquisition by 71% (95% CrI 30-97%). Using these as inputs in the population-level modelling, the projected impact on HIV incidence is shown in Figure 2.

Table 1 presents the projected impact of microbicide introduction under a number of scenarios. In the main intervention scenario with mean efficacies (30% uptake achieved over 10 years, gel used in 72% of sex-acts and no reduction in condom use), we predict that the gel could lead to a 12.5% (95%CrI 11.5-12.7%) relative reduction in HIV incidence and a 4.9% (95%CrI 4.7%-5.5%) relative reduction in HSV-2 incidence by year 15. This is a reduction in HIV incidence in the whole population from 0.56 (95%CrI 0.48-0.72) per 100 person-years to 0.49 (95%CrI 0.42-0.64) per 100 person-years, and a reduction in HSV-2 incidence from 7.4 (95%CrI 6.4-7.5) per 100 person-years to 7.1 (95%CrI 6.0-7.1) per 100 person years.

**Table 1:** Projected impact from microbicide intervention*

	**Mean efficacies**		**Low efficacies**		**High efficacies**	
	**(54% HIV efficacy and 71% HSV-2 efficacy)**		**(8% HIV efficacy and 30% HSV-2 efficacy)**		**(83% HIV efficacy and 97% HSV-2 efficacy)**	
	**Trial consistency of gel use (72%)**	**Reduced consistency of gel use (used in 50% of sex-acts)**	**Trial consistency of gel use (72%)**	**Reduced consistency of gel use (used in 50% of sex-acts)**	**Trial consistency of gel use (72%)**	**Reduced consistency of gel use (used in 50% of sex-acts)**
HIV impact projections*:						
Percentage reduction in population HIV incidence by year 15	12.5%	8.7%	2.0%	1.4%	19.0%	13.3%
	(11.5-12.7%)	(8–8.8%)	(1.8-2.0%)	(1.3-1.4%)	(17.6-19.3%)	(12.3-13.5%)
Cumulative number of HIV infections averted over 15 years	55,366	38,382	8,661	6,011	85,026	58,900
	(49,309-58,173)	(34,132-40,297)	(7,708-9,255)	(5,348-6,422)	(75,845-89,447)	(52,447-61,890)
Cumulative number of HIV infections averted per 100,000 population	970	672	152	105	1,489	1,031
	(864-1,019)	(598-706)	(132-162)	(94-112)	(1,328-1,560)	(918-1,084)
Number of sex-acts protected by gel per HIV infection averted	1,317	1,896	8,372	12,060	861	1,239
	(1,266-1,481)	(1,853-2,133)	(8,176-9,257)	(11,779-13,338)	(839–966)	(1,209-1,392)
HSV-2 impact projections*:						
Percentage reduction in population HSV-2 incidence in year 15	4.9%	3.4%	1.6%	1.1%	7.0%	4.8%
	(4.7-5.5%)	(3.2-3.7%)	(1.5-1.8%)	(1.0-1.2%)	(6.8-7.9%)	(4.6-5.4%)
Cumulative number of HSV-2 infections averted over 15 years	83,997 (76,312-88,153)	57,343 (52,139-60,191)	27,181 (24,537-28,617)	18,723 (16,930-19,711)	121,846 (110,559-127,721)	82,662 (75,047-86,655)
Cumulative number of HSV-2 infections averted per 100,000 population	1,471	1,004	476	327	2,133	1,447
	(1,336-1,543)	(913-1,054)	(430-501)	(296-345)	(1,936-2,236)	(1,314-1,518)
Number of sex-acts protected by gel per HSV-2 infection averted	868	1,269	2,668	3,872	601	883
	(831-977)	(1,213-1,428)	(2,566-2,994)	(3,721-4,346)	(575-677)	(845-994)

*Assuming gel only used by HIV-negative women, and levels of condom use are maintained after microbicide introduction.

These reductions in incidence translate into averting 970 (95%CrI 864–1,019) HIV infections and 1,471 (95%CrI 1,336-1,543) HSV-2 infections per 100,000 population (Table 1) over 15 years. In this main scenario, one HIV infection and one HSV-2 infection is averted for every 1,317 (95%CrI 1,266-1,481) and 868 (95%CrI 831–977) microbicide protected sex-acts, respectively.

The impact projections are dependent upon the assumptions made regarding gel’s efficacy, consistency and uptake, and the degree to which condom use may or may not be affected by gel introduction. For example, if the gel is used in 50% rather than 72% of sex-acts, then the number of HIV infections averted is reduced by 30% in relative terms.

Figure 2 shows how the HIV impact is affected by a number of modelling assumptions; the dark bars represent the main intervention scenario. Gel efficacy is very important, with a very highly efficacious gel potentially reducing HIV incidence by 19.0%, while a gel with poor efficacy provides little population protection. Higher levels of uptake can have an important impact with a doubling of uptake resulting in a doubling of impact. There is also a 25% relative reduction in impact if condoms are used in 5% fewer sex-acts but uptake and gel use is maintained (Figure 2).

Interestingly the model projections suggest the HSV-2 efficacy of the gel contributes little to HIV-impact. Even without any HSV-efficacy, the relative reduction in HIV incidence is still projected to be 12.0% after 15 years for the main intervention scenario compared to 12.5% if the gel is 71% HSV-2-efficacious. However, the HIV efficacy has an important influence on HSV-2 incidence: the relative reduction in HSV-2 incidence is projected as 4.9% with 54% HIV efficacy versus 3.5% if the gel has no HIV efficacy.

### Microbicide cost effectiveness and threshold prices

Table 2 presents the cost-effectiveness of microbicide introduction. For the main intervention scenario (30% uptake, 72% gel use, 54% HIV efficacy) with two gels per sex-act, three HIV tests and collection visits per year (after the initiation visit), the cost per DALY averted is $297 at the price of $0.25 per dose. At current trial product costs of $1.60, the cost per DALY averted would be $2,660. At the lowest production cost, $0.17 using cardboard applicators and large scale production, the cost per DALY averted would be as low as $157. This variation emphasises the large contribution of the product in the total programme costs, ranging from 42% at the lowest price ($0.17) up to 87% at the highest price ($1.60). At $0.25, the product accounted for 51% of total programme costs, while HIV counselling and testing accounted for 38% of costs: each extra HIV test would increase the cost-effectiveness ratio by 36%. The health facility costs of visits for adherence counselling and gel collection are relatively low, contributing only 9% to programme costs: changes in collection frequency provide little savings or extra costs. However, a more integrated approach, with adherence counselling and gel distribution during the HIV testing visit, may be more attractive to clients and is more efficient, at $221 per DALY averted. For microbicide introduction to be equally cost-effective as VMMC, product costs would need to drop below our lowest estimated production cost, to $0.12 for the base scenario. By integrating gel collection and HIV testing, or by reducing the HIV tests to two per year, the cost threshold would just reach or surpass our lowest projected product cost.

**Table 2:** Cost-effectiveness and threshold prices under varying programmatic assumptions (2012 US$)*

		**Cost per DALY averted at $0.25 per dose**				**Microbicide threshold prices where $/infection averted (IA) = VMMC (~$1000/IA)**	
		**2 doses per sex-act**		**1 dose per sex-act**		**2 gels per sex-act**	
Baseline programme: 3 annual HIV tests, 3 gel collection visits		$297				$0.12	
*Variations to distribution services*							
HIV tests per year	2	$190	-36%	-$29	-137%	$0.19	49%
	4	$405	36%	$186	137%	$0.06	-49%
	6	$619	108%	$401	411%	Cannot be achieved	
Adherence counselling and gel collection visits	2	$272	-9%	$53	-32%	$0.14	12%
	6	$374	26%	$155	97%	$0.08	-35%
Integrated adherence counselling and gel collection into HIV testing (3)		$221	-26%	$2	-97%	$0.17	35%

*Assuming 30% uptake after 10 years and trial consistency of gel use and mean imputed efficacy estimates, 10% product wastage during distribution, and a 3% discount rate on costs and effects.

We also model the cost-effectiveness and threshold prices for variations to gel uptake, use consistency, and HIV efficacy, linked with the different impact scenarios (Table 3). A five percentage point absolute decrease in the percentage of sex-acts in which a condom is used would increase the cost to $548 per DALY averted (i.e. worsen the cost-effectiveness), and a reduction in condom use of 10 percentage points would generate a cost of $1,219 per DALY averted. A more efficacious gel could cost as much as $0.33 per dose to be equally cost-effective as VMMC, and at $0.25 would generate an cost saving, indicated by the negative cost per DALY averted (-$14). Consistency of gel use (adherence) is also important, where only using gel in half the sex-acts reduces effectiveness by more than the reduction in costs, with cost of $468 per DALY averted. This worsening is attributable to maintaining the annual fixed costs per facility and woman, while reducing gel use, and emphasises how important user behaviour will be in the products’ ultimate cost-effectiveness. Uptake, however, has a relatively small impact on the break-even point: doubling uptake just increases the threshold price by 5%, with both the fixed cost of reaching women and their gel use being reduced.

**Table 3:** Cost-effectiveness and threshold gel prices under variations to introduction scenario assumptions and sensitivity analysis [2012 USD]

***Variations to introduction scenario***								
	**Reduction in condom use**	**Coverage**	**Consistency of gel use**	**HIV efficacy**	**$/DALY averted**	**% change**	**Threshold price**	**% change**
Baseline	0%	30%	72%	54%	$297		$0.12	
	5%	30%	72%	54%	$586	97%	$0.03	-74%
	10%	30%	72%	54%	$1,219	310%	cannot be achieved	
	0%	60%	72%	54%	$285	-4%	$0.13	5%
	0%	60%	50%	54%	$468	58%	$0.03	-78%
	0%	30%	72%	83%	-$14	-105%	$0.33	166%
*Cost sensitivity analysis*								
1 dose per sex-act					$78	-74%	$0.25	100%
Discount rate 0%					*-$243*	-182%	$0.43	244%
Discount rate 6%					$525	77%	cannot be achieved	
ART costs 10% lower					$344	16%	$0.10	-21%
ART costs 25% lower					$413	39%	$0.06	-53%
All other input costs 25% higher					$402	35%	$0.06	-48%
All other input costs 25% lower					$193	-35%	$0.18	48%
Input +25%, ART-10%					$448	51%	$0.04	-69%
Input +25%, ART-25%					$518	74%	-$0.00	-100%
ART coverage 80%					$163	-45%	$0.24	90%

A sensitivity analysis of key cost assumptions is presented in the bottom half of Table 3.

If a single dose regimen were found, the price could be as high as $0.25, in line with expectations of production costs at scale. Though cost-effectiveness analyses have traditionally assumed an international discount rate of 3%, more recent analyses have started using local discount rates to account for local time preferences which then better represent local decision making. The South African official discount rate in 2012 was 5.5%. A 6% discount rate has a very large impact on the cost-effectiveness: $525 per DALY averted at 6% versus cost saving when discounting is removed in the costs. As the cost of ART provision has decreased dramatically over the past decade, we also explore the trade-off between treatment and prevention for lower ART provision costs. If ART costs were to drop by either 10% or 25%, the cost-effectiveness of providing microbicides would worsen, increasing to $413 per DALY averted. To account for variations in programme costs we explored changes to all other input prices by +/- 25%, which would change cost-effectiveness ratio by -/+ 35%, respectively. In the most conservative case, where programme costs were increased by 25% and ART costs decreased by 25%, the cost DALY averted is estimated at $518. If ART coverage were expanded from 52% to 80%, in line with the 2016 national target [37], then the threshold price of microbicides could be $0.24. Though under most circumstances the explored microbicides are unlikely to be as cost-effective as VMMC, they would however reach a complementary target group (women) to which VMMC can never provide direct protection.

## Discussion and conclusions

We have used detailed mathematical and economic modelling to estimate the impact and break-even price of tenofovir gel as introduced and distributed in urban South Africa. Our findings suggest that, for the main scenario considered, gel introduction could be an important addition to existing HIV prevention efforts, leading to a 12.5% (95%CrI 11.5-12.7%) relative reduction in HIV incidence and a 4.9% (95%CrI 4.7-5.5%) relative reduction in HSV-2 incidence over 15 years. The impact on HSV-2 is less than the impact on HIV, despite the higher efficacy against HSV-2, due to higher risk of HSV-2-infection for women.

Our findings suggest that the introduction of an effective gel could be cost saving at reasonable gel prices, in particular as ART access is expanded, and highly cost-effective even at current gel prices.

Indeed, using plausible assumptions about the full costs of programme delivery, we predict that the introduction of an HIV efficacious gel would be highly cost-effective ($297 per DALY averted) at a price of $0.25, which is within the cost range ($0.17-$0.27) at large scale production of 100 million applicators [36]. In our model this scale of distribution is achieved from year 9 onwards. If South Africa reaches its target of 80% on ART or a single dose regimen is found to be effective, we can expect microbicides to be around the same cost per HIV infection averted as VMMC.

Even at current FACTS 001 trial production prices ($1.60) [36], the incremental cost per DALY averted would be around $2,660, which is well below the commonly applied cost-effectiveness threshold of 1*GDP ($7,610 for South Africa) [42].

Using the trial estimates of 72% adherence, we impute that the HIV and HSV-2 per sex-act efficacies of tenofovir gel are 54% (95% CrI 8-83%) and 71% (95%CrI 30-97%), respectively. Although these estimates are based upon the trial measures of gel adherence reporting, we considered that these are reasonably reliable, as gel applicator returns were found to correlate well with vaginal tenofovir concentration measured in a pharmacokinetics sub-study [43]. We are also reassured about the validity of our imputed efficacy estimates because a secondary analyses of the effectiveness among ‘high adherers’ in the trial (i.e. women who used both doses of the gel in over 80% of sex-acts) found that the risk of HIV acquisition in this population was reduced by 54% (95%CI 4-80%) [5]. However, the wide bounds on the imputed efficacy estimates of tenofovir gel, due to the wide confidence intervals on the trial effectiveness estimates, lead to a wide range of population-level impact estimates predicted by the model.

This analysis builds upon our previous modelling of the potential impact of microbicide introduction in different settings [44-48], as well as the work by Williams et al. [48], Verguet et al. [39] and Walensky et al. [49-51]. Additional strengths here include that the dynamic model is carefully parameterised and rigorously fitted to a specific setting over multiple time points, including fitting to HIV and STI infection status by sex, as well as incorporating the HSV-2-efficacy of tenofovir found in CAPRISA 004 and the related bi-directional interaction between HSV-2 and HIV and the dynamic effects of gel on the HIV and HSV-2 epidemic. Walensky et al. [50] included only the first generation of HIV infections averted. Numerous programme implementation components were also included in our analysis, such as training, mass media and facility distribution costs, to provide a more comprehensive approach to costs than comparable studies to date. However, we did not account for potential toxicity or ART resistance as in Walensky et al. [50], because the concerns in this area are limited, as the authors themselves acknowledge. Moreover, the scenarios that we have modelled are less optimistic than those considered previously [49]. This study makes lower assumptions regarding the likely coverage that can be achieved, more optimistic assumptions about the impact of ART on life expectancy and the discounting of future DALYs averted (an sometime omitted step [51]. Consequently, we produce a far more conservative estimate of impact and cost-effectiveness.

Our modelling stresses the importance of maintaining condom use following microbicide introduction, to avoid large reductions in impact and cost-effectiveness. This issue is particularly of concern if condom use is already high [48], and for a microbicide with lower efficacy, as concomitant reductions in condom use could even lead to increases in the overall number of infections [48]. Similarly, our analyses highlight the degree to which the costs of gel introduction will be affected by programme costs, including the unit cost of gels, and the required frequency of HIV testing. For this reason, further research is needed to identify the optimal spacing between HIV testing, with the aim of achieving an appropriate balance between what may be required to minimise the potential for resistance and be feasible for women and health systems. Similarly, research to explore whether a single dose of tenofovir-containing gel, applied prior to sex, could be effective at reducing a woman’s risk of acquiring HIV is needed [52], to help reduce the product costs, as well as make gel use less burdensome for women.

The fact that 17% of VOICE participants reported engaging in anal intercourse during the 3-month period prior to enrolment highlights an important area for future modelling work [9]. For such modelling, data is needed from different sites and population groups on the frequency of anal sex over a specific timeframe (e.g. past 3 or 6 months), and whether a condom and/or gel is used during the last anal sex-act.

Although it is tempting to draw broader, national or regional conclusions from this analysis, our model projections may not be generalisable to other settings with different epidemic and behavioural profiles and different price levels. However, the impact we predict compares favourably with previous modelling of a hypothetical microbicide introduced into an area within Gauteng [19], and with modelling studies of the impact of male circumcision on HIV incidence in various settings of sub-Saharan Africa [53-55], matched to the same intervention assumptions made here [56].

The results are also highly sensitive to the discount rate used. Our central estimate used a 3% discount rate as commonly applied in international studies. However, when using the upper bound of 6%, just above the South African national discount rate of 5.5%, the cost-effectiveness of microbicides would worsen dramatically by 77%. Although not a realistic price to expect during mass-production, the programme’s incremental cost-effectiveness, at the current product cost of $1.60 per dose would still be highly cost-effective at $2,825 per DALY averted even using a 6% discount rate.

Some have questioned the viability of tenofovir gel given recent disappointing evidence of low user adherence in the VOICE trial. That users have a difficult time with adherence, however, should come as no surprise. Inconsistent adherence is the norm for both medications and any prevention methods that are user-controlled. Nonetheless, every indication is that the South African government and international donors are prepared to seek licensure for tenofovir gel should the results of the CAPRISA trial be sustained in the on-going FACTS 001 trial. Given that policymakers will soon face a key decision point regarding the possible introduction and scale up of tenofovir gel, the findings of this article are highly relevant. Despite intuitions to the contrary, it appears from the results of VOICE, FEMPREP, and other trials of daily oral or vaginal prophylaxis, that women seem better able to adhere to the coitally-dependent BAT-24 regimen than daily use.

Moreover, even if the attention of the wider field has shifted towards long-acting modalities such as the vaginal ring and injection, these products are still many years from licensure and there is undoubtedly benefit of beginning to prepare consumers for using ARVs to prevent infection. There will always be a market for methods like the vaginal gel that will likely not require on-going HIV testing and that can be positioned as a sexual lubricant with additional HIV fighting powers. Though highly cost-effective prevention options are available for men (VMMC and male condoms), there is still a great need to seek coitally-dependent options that women can initiate, such as microbicides, in whichever formulation.

This study has used epidemiological and cost modelling to estimate the impact of microbicide introduction among women of the general population in the Gauteng Province in South Africa. We used highly conservative assumptions to estimate gel impact and found that microbicides have the potential to significantly reduce HIV incidence among women in Gauteng. This is the most comprehensive model of the costs of microbicide introduction available in the literature to date and, even under the very conservative assumptions this analysis, estimated that microbicide introduction is very likely to be highly cost-effective. However this analysis also highlighted the importance of continued strong condom distribution and counselling programmes to maintain condom use and the need for identifying innovative approaches for supporting microbicide adherence.

## Endnotes

^a^Adherence estimates based on applicator returns for the remaining 884 women indicate that, on average, 72.2% (median = 60.2%) of self-reported sex-acts in the last 30 days were covered by two doses of gel.

^b^It should be noted that condom use outside the trial setting is lower and our approach will therefore underestimate the impact of microbicides on HIV incidence.

^c^The 6% discount rate is consistent with the South African central bank discount rate of 5.5% [57].

## Abbreviations

ART: Antiretroviral therapy; CI: Confidence interval for data estimates; Crl: Credibility interval for model projections – defined as the 2.5 to 97.5% percentile range; DALY: Disability adjusted life year; DfID: Department for International Development; FSW: Female sex workers; HIV: Human immunodeficiency virus; HSV-2: Herpes simplex type 2; ICER: Incremental cost-effectiveness ratio; IPM: International Partnership for Microbicides; MDP: Microbicide development programme.

## Competing interests

I have read the journal’s policy and have the following conflicts: AF, FTP, AC, PV, CW received a grant from IPM in the initial development of these models. At the time, TM was employed by IPM. IPM aims to promote the development and delivery of safe and effective microbicides around the globe. LH was previously the director for the Global Campaign for Microbicides.

## Authors’ contributions

Conceptualised the study: AF, PV, FTP, TM, AC, LH,CW; Developed the model: PV and AF; Undertook the impact modelling: AC and AF and PV; Undertook the cost modelling: FTP; Wrote the first draft of the manuscript: AF, FTP; Contributed to the writing of the manuscript: LH, AC, GMR, SDM, TM, HR, PV, CW; Criteria for authorship read and met: AF, FTP, AC, LH, GMR, SDM, TM, HR, PV, CW. Agree with manuscript results and conclusions AF, FTP, AC, LH, GMR, SDM, TM, HR, PV, CW. All authors read and approved the final manuscript.

## Pre-publication history

The pre-publication history for this paper can be accessed here:

http://www.biomedcentral.com/1471-2334/14/14/prepub

## Supplementary Material

Additional file 1: Table S1 Average additional life expectancy and reduction in transmission probabilities for the population attributable to ART treatment. **Table S2.** Behavioural and demographic parameters used in the model for Gauteng Province. **Table S3.** Biological parameters used in the model for Gauteng Province. **Table S4.** Data used in model fitting: Prevalence of Sexually Transmitted Infections – Gauteng/South Africa. **Table S5.** Cost inputs, assumptions and sources. Click here for file
