# Supplementary material for: Cost-effectiveness of tenofovir gel in urban South Africa: model projections of HIV impact and threshold product prices
Source: BMC Infect Dis. 2014 Jan 9;14:14. doi: 10.1186/1471-2334-14-14 (PMC3899035; doi:10.1186/1471-2334-14-14)
Supplement: Additional file 1: Table S1 — Average additional life expectancy and reduction in transmission probabilities for the population attributable to ART treatment. Table S2. Behavioural and demographic parameters used in the model for Gauteng Province. Table S3. Biological parameters used in the model for Gauteng Province. Table S4. Data used in model fitting: Prevalence of Sexually Transmitted Infections – Gauteng/South Africa. Table S5. Cost inputs, assumptions and sources. [file 1471-2334-14-14-S1.docx]

##### Additional file

##### Technical description of model

The model is a development of a previous model developed by Peter Vickerman and described in Vickerman, *et al.* (2006 and 2010) [1-2]. The original model simulated the transmission of HIV and two curable STIs resulting from heterosexual contacts between a number of sub-populations within the male and female population. The model has been adapted to include the heterosexual transmission of Herpes simplex virus type 2 (HSV-2), one of the curable STIs has been adapted to mimic the natural history of *Treponema pallidum* (syphilis).

Individuals enter the simulated population when they become sexually active, and have a specified level of sexual activity (in terms of whether or not they engage in commercial sex and the different types of partnerships they form), with the rates of partner change, frequencies of sex and levels of condom use determined using setting specific behavioural data. FSW and clients stop selling and buying sex at a set rate, enter the non-commercial population subgroups, and are replaced by an equal number becoming FSW/clients, but spread proportionally over all infection classes. Individuals leave the population due to natural death or AIDS-related morbidity/mortality.

The model was formulated in *C* programming language and consists of a set of deterministic ordinary differential equations that describe the movement of individuals between discrete subgroups – based upon their sex, sexual behaviour, STI and HIV infection status. These are described in turn below. Established mathematical techniques were used to estimate how HIV and other STIs spread between the subgroups over time [3-5].

In the model, the population is divided by sex (*r=0* and *r=1* denotes males and females respectively) into a number of sub-groups with four different levels of sexual activity (*j=0*, sexually inactive, *j=1*, just has long-term ‘main’ sexual partners, *j=2*, has main and casual sexual partnerships, j=3, has main, casual and commercial sexual partnerships). The population is also divided into four HSV-2 infection stages (i = 0 to 3, with 0 being uninfected; 1 being the initial primary infection stage; 2 being the asymptomatic stage and 3 being the symptomatic recurrence phase). The subscripts r’,p,k denote the sex, sexual activity and HSV-2 infection status, respectively, of a partner of an individual of sub group r,j,i.

As with other HIV compartmental models, each behavioural sub-group is divided into those that are susceptible to HIV infection (*x*), those that are recently HIV infected and in the initial high viraemia phase (*h*), those who have progressed into the low-viraemia phase (*y*) and those with symptomatic high viraemia (z). Those with AIDS are chronically ill, and are assumed to

cease being sexually active. New individuals enter the susceptible population at a fixed per capita recruitment rate (*Λ_r_*) equivalent to the sum of those leaving the model through HIV related and unrelated mortality and migration. The overall number in the modelled population is represented by the term n. Susceptibles become infected with HIV at a per capita rate that is determined by the per-capita risk associated with their sexual behaviour (π_sex_). When a susceptible becomes infected with HIV they are initially highly infectious (for an average period *1/ν*). They then enter a long period of low infectivity (average duration *1/*$\Omega$) before entering the symptomatic high infectivity phase (for an average period 1/$\Psi$). In the model, individuals remain in the population until they cease sexual activity due to AIDS, die or migrate out of the population. The non-HIV related movements out of the population are summed and represented by the parameter.

Female sex workers continue sex work for a period (average duration 1/η_rj_, for r=1 and j=3) before returning to the general population and are replaced by new female sex workers from the sexually active general population. Similarly, clients remain as clients for a period (average duration 1/η_rj_, for r=0 and j=3) before returning to the general population and being replaced by new clients from the sexually active general population. The parameter η_rj_ is zero for j=0, 1 and 2. The female sex workers and clients only return to the corresponding infection categories amongst the sexually active individuals that have just main (j=1) or main and casual partnerships (j=2), with the proportion going to each being dependant on the relative size of each groups. In addition, the female sex workers and clients are only replaced by individuals from the j=1 and j=2 behavioural categories, but from all HIV and HSV-2 infection states, with the number from each category being depending on the relative number in that category. Equation 1 describes the HIV infection dynamics amongst the population including the population movements into and out of the FSW and client groups:

Equation 1

where *δ_jp_* equals one if *j=p* and zero otherwise. The probability that a susceptible becomes HIV infected per unit time from unprotected sex (π_sex_) is one minus the probability of not getting infected over this time. The probability of not becoming infected per unit time is the product of the probabilities of not being HIV infected from any sexual act with any sexual partner from each behavioural sub-group. If we let *d0_rj_*, *d1_rj_* and *d2_rj_* denote the total number of main, casual and commercial sexual partners that an individual in sub-group *[r,j,i]* has per unit time, then mathematically, the probability that a susceptible becomes HIV infected from their sexual partnerships (*π_sex_*) per unit time is given by:

Equation 2

where the function *ϕ0_rjipk_*  *ϕ1_rjipk_*  and *ϕ2_rjipk_*  are the probabilities that a susceptible in sub-group *[r,j,i]* will become HIV infected per unit time from a main, casual or commercial sexual partnership with an individual in sub-group *[r’,p,k]*, and *ρ0_rjipk_*, *ρ1_rjipk_* and *ρ2_rjipk_* are the probabilities that individuals within sub-group [r,j,i] form main, casual or commercial sexual partnerships with individuals from [r’,p,k], with different levels of sexual activity (j and p) and HSV-2 status (i and k).

Two components make up the mixing function *ρ0_rjipk_*. Firstly, an individual has a probability ‘*є’* that they will choose a main partner of the same sexual activity, and otherwise their main partnerships are chosen depending on the number of main partnerships provided by different sub-groups. The probability *є* determines how assortative or random the mixing is, with 1 being complete like with like assortative mixing and 0 being random mixing. The mixing function *ρ0_rjipk_,* has the following form:

$\rho0_{rjipk}=\left(1-\varepsilon\right)\frac{d0_{r'p}n_{r'pk}}{\sum_{\forall p,k} d0_{r'p}n_{r'pk}}+\varepsilon\delta_{jp}\frac{d0_{r'p}n_{r'pk}}{\sum_{\forall k} d0_{r'p}n_{r'pk}}$,

Equation 3

The form and derivation of the *ρ0_rjipk_* function is a development of the formulation by Garnett and Anderson [46]. The product *d0_rj_ρ0_rjipk_* then gives the total number of main sexual partnerships an individual from sub-group *[r,j,i]* forms with those from sub-group *[r’,p,k]*. The functions *ρ1_rjipk_* and *ρ2_rjipk_* and calculated similarly except mixing is assumed to be random (proportional to the number of casual or commercial partnerships provided by that group) and *ρ1_rjipk_*>0 only when j>1 (only individuals in classes j=2,3 have casual partnerships) and *ρ2_rjipk_*>0 only when j>2 (only individuals in classes j=3 have commercial partnerships).

The parameter ϕ0_rjipk_ is the product of the probability that an individual from that behavioural and HSV-2 infection state is infected and the probability that the infected main partner transmits HIV to the susceptible individual per unit time (defined as D0_rjipk_). The probabilities of HIV transmission between a susceptible and an infected individual per unit time are estimated using a Bernoulli formulation based on Weinstein *et al*. (1989) [[5](#_ENREF_5)]. If in each unit of time, an individual in sub-group [r,j,i] has m_j_ sex-acts with each main sexual partner; f is the probability that a condom is used per sex-act; e is the per sex-act efficacy of the condom; β_r_ is the probability of HIV transmission per sex-act from sex r’ to sex r; α_s_ is the extent to which STI co-infection of either partner increases the probability of HIV transmission, and α_hi1_ and α_hi2_ are the extent to which the initial and symptomatic high viraemia phases increase the probability of HIV transmission, then D0_rjipk_ can be written:

Equation 4

where y^s^ denotes the number infected with an STI and

$$\begin{matrix} \Phi=\left[1-\beta_{r}\left(1-ef\right)\right]^{m_{j}} \\ \Gamma=\left[1-\alpha_{hi1}\beta_{r}\left(1-ef\right)\right]^{m_{j}} \\ \Theta=\left[1-\alpha_{hi2}\beta_{r}\left(1-ef\right)\right]^{m_{j}} \end{matrix}$$

$$\begin{matrix} E=\left[1-\alpha_{s}\beta_{r}\left(1-ef\right)\right]^{m_{j}} \\ H=\left[1-\left(\alpha_{s}+\alpha_{hi1}-1\right)\beta_{r}\left(1-ef\right)\right]^{m_{j}} \\ \Omega=\left[1-\left(\alpha_{s}+\alpha_{hi2}-1\right)\beta_{r}\left(1-ef\right)\right]^{m_{j}} \end{matrix}$$

Equation 5

Here the variables Φ, Ε, Γ, Θ, Ω and Η denote the probabilities that a susceptible person, who is having sex with an HIV infected person, does not become HIV infected per unit time in the following different situations; neither partner has a particular STI and the HIV infected partner is not in the initial high viraemia phase (Φ); at least one partner has an STI and the HIV infected partner is not in the initial high viraemia phase (Ε); neither partner has an STI but the HIV infected partner is in the initial high viraemia phase (Γ); neither partner has an STI but the HIV infected partner is in the symptomatic high viraemia phase (Θ); at least one partner has an STI, and the HIV infected partner is in the symptomatic high viraemia phase (Ω); at least one partner has an STI, and the HIV infected partner is in the initial high viraemia phase (Η). Equations 4 and 5 are extended to include the possibilities of each partner having a curable STI or syphilis or HSV-2, but are not shown here for simplicity. The overall cofactor for when two STIs are present or when in a high viraemia phase of HIV is additive.

The corresponding probabilities ϕ1_rjipk_ and ϕ2_rjipk_ (probabilities that a susceptible will become HIV infected per unit time from their casual or commercial partnerships), and D1_rjipk_ and D2_rjipk_ (probabilities that a susceptible will become HIV infected per unit time from their infected casual or commercial partnerships) are calculated in exactly the same way except that there are different levels of condom use for casual and commercial sexual partnerships and only certain individuals have casual (j=2,3) and commercial partnerships (j=3).

### STI dynamics

The model also simulates the transmission of syphilis, HSV-2 and a generic bacterial STI (representing *Neisseria gonorrhoeae* (Ng) and *Chlamydia trachomatis* (Ct)) between sexual partners. In brief, once individuals are infected with Ng/Ct, they become susceptible again at a fixed rate. Once individuals are infected with HSV-2, they remain infected for life. They first enter the initial infection stage, then progress to asymptomatic infection from which they repeatedly have short symptomatic recurrences [6-7]. When individuals are infected with syphilis, they enter the primary/secondary phase of infection, then progress to latent infection where they remain, if not treated. If syphilis cases are treated in the primary/secondary infection stage, then they become susceptible, whereas they become temporarily immune [8-10] if they are treated during latent infection.

For the bacterial STI an SIS model structure was adopted, whereas for syphilis there are four classes: susceptible, primary and secondary infected, latent infected and resistant. Both HIV susceptible and HIV infected individuals can acquire an STI. The per capita probability of STI transmission per unit time (*π_s_*) is analogous to the per capita probability of HIV infection per unit time (*π_sex_*) (*equation 2*) but only primary and secondary syphilis cases are assumed to be infectious. If the probability of STI transmission per sex-act is *β_std_*, and *y^s^* is the number infected with an STI (only those in the primary or secondary stages for syphilis), then for each partnership the probability that a susceptible is infected with a STI per unit time (defined as *φ^s^_rjipk_*) is:

Equation 6

If we let *x^s^* denote the number susceptible to an STI, and *y^s^* the number of infecteds, then the transmission dynamics of the SIS STI can be described using the following set of deterministic differential equations:

Equation 7

For this we assume that individuals remain infected for a fixed period of time (*1/μ_r_*), and then become susceptible to the STI once more, and the parameter $\Psi$ is the rate at which susceptibles and infecteds leave due to HIV morbidity (acquiring AIDS) because a proportion of the individuals in sub-group [r,j,i,] will be in the symptomtic HIV phase.

For syphilis, the dynamics are similar to those described for the first STI (equation 7) except there are three stages of infection, primary (*y^tp^*), latent (*l^tp^*) and resistant or immune (*r^tp^*) and individuals susceptible to syphilis are denoted by *x^tp^*.

For syphilis, if individuals are treated when in the primary or secondary stage (at a rate $\omega_{rj}$) then they become susceptible again, whereas if they are treated in the latent stage (at a rate $\sigma_{rj}$) then they are assumed to become resistant to infection for a certain duration ($1/\upsilon$). If individuals are not treated then they stay in the primary or secondary stage for an average duration $1/\theta$and then enter the latent phase where they remain until treated. The differential equations in equation 8 describe the syphilis model:

Equation 8

**HSV-2 dynamics**

HSV-2 infection states are modelled as an index (i) of all other state variables. This means that HIV and HSV-2 are modelled in a way that all possible co-infection states are modelled explicitly. The model structure used for HSV-2 is described in detail by Foss *et al.* (2009 and 2011) with there being four HSV-2 infection states (i=0..3) [6-7]. The probability that a susceptible is infected with a HSV-2 per unit time (defined as $\phi_{rjipk}^{h}$) uses the same formulation (equation 6) as for the other STIs except that there are different transmission probabilities for primary, latent/asymptomatic and recurrent HSV-2.

HSV-2 infection is modelled in a similar manner to the other STIs with slight modifications. The index i denotes the stage of HSV-2 infection and stratifies all behavioural and HIV states. The susceptible population (i=0) becomes infected with probability . After infection individuals move into a short initial/primary infection stage (denoted by i=1) with average duration 1/$\gamma$. From this initial infection stage individuals move into the latent/asymptomatic low level infectious shedding stage (i=2). Individuals in the latent stage can move in to a symptomatic recurrent phase (i=3) at rate $\varsigma$which differs for those who are HIV positive, before returning back to the asymptomatic phase at an average rate $\varpi$. Individuals with HSV-2 are assumed to remain infected for life. The differential equations for HSV-2 for individuals in a generic HIV state ‘q’ are as shown below (equation 9). With $\Psi$only being present if the generic HIV state is the symptomatic z state and the replacement of susceptibles only occurring in the HIV susceptible state unless a portion of the incoming population are assumed to be HIV or HSV-2 infected.

Equation 9

**Modified susceptibility / infectivity dependant on STI infection status**

The model is structured so that the degree of infectivity of those infected and/or susceptibility to infection are modified according to the individual’s infection status for other infections. Susceptibility to HIV infection and HIV infectivity are increased in those infected with syphilis (only primary and secondary syphilis), bacterial STI or HSV-2 (with different cofactors for each stage of HSV-2). There is also a different rate of asymptomatic and symptomatic shedding of HSV-2 for those co-infected with HIV. The infectivity of HSV-2 is also increased if the individual is co-infected with HIV, and the susceptibility to infection with HSV-2 is increased in those infected with HIV.

**Approach to modeling the effect of ART**

The effect of ART in the model was implemented at the entire population level (increase in overall life expectancy and reduction in overall HIV transmission probability). We chose to default to overestimating the effects of ART rather than underestimating its effects so that the microbicide impact estimates would be conservative. To that end, we chose parameters to calculate the effect of ART that were in excess of current reported values.

**Adjusting the Transmission Probability for ART Coverage**

Using the numbers of adults on ART for Gauteng [11], the annual HIV prevalence for Gauteng/South Africa, and the population size, we are able to calculate the proportion of HIV-infected people receiving ART from 2002 to 2008. For future years we assumed that those who had been infected with HIV for more than three years were eligible for ART and 80% of these would receive ART by 2016 (following South Africa’s treatment coverage targets{South African Department of Health, 2012 #196}). Three years from initial HIV infection corresponds to half-way through the low-vireamia phase, which means that the ART-eligible population consists of half of the population in the low-vireamia phase, all those in high-vireamia pre-AIDS phase and everyone with AIDS. Assuming 80% of those eligible receive (and adhere to) ART coverts, in this way, to 60% of all those HIV-infected receiving and adhering to ART by 2016, after which time we model ART coverage to remain constant.

Between 2008 and 2016 we assumed a linear increase in ART coverage.

To enable estimation of the effect of ART on transmissibility we assumed that HIV transmission probability when treated is 1% of that for those untreated. While we acknowledge that current estimates of the reduction are 4% though decrease to 0% when only linked transmission was considered [12]*),* we chose this value of 1% to be as conservative as possible whilst still allowing for some possibility of transmission events whilst on ART (as in Cox et al, 2011 [13])*.* We can then calculate an average reduction in the transmission probability for the entire population. This is calculated as a weighted average as follows.

Relative reduction in transmission probability = (Number of HIV positive not receiving ART + number of HIV positive receiving ART*1%)/total number of HIV positive

See Supplementary Table 1 below for transmission probability multipliers.

**Additional increase in life expectancy due to ART coverage**

The duration of HIV is increased for the entire population by assuming that those with HIV and receiving ART have a normal life expectancy. The increase in life expectancy experienced by those receiving ART treatment was incorporated into the model by averaging the increase attributable to the reported yearly coverage of ART across the entire population. The life expectancy used in the model, derived from Statistics South Africa (2004)[14], was assumed to include HIV-related mortality but no significant effect of ART treatment. Any effect of existing ART treatment in this reported life expectancy would serve to make our implementation of the effects of ART an overestimate and therefore provide a conservative assumption about microbicide impact. Treatment with ART was assumed to add an additional 20 years to the life expectancy for those who receive treatment compared with those not receiving treatment. Current estimates of the increase in life expectancy afforded by ART treatment in South Africa report an 11.3 year gain[15]. A 20 years gain in life expectancy was chosen to provide a conservative estimate of microbicide impact. The calculations were made for each year beginning in 2001 and using the ART coverage levels reported in Adam & Johnson [11] and the assumption that ART coverage would plateau at 60% of HIV infected individuals by 2016. The additional life expectancy was calculated as (number receiving ART * additional life expectancy)/total population.

The resulting averaged increase in life expectancy, and transmission probability, applied to the entire population in the model are detailed in the table below.

**Table S1: Average additional life expectancy and reduction in transmission probabilities for the population attributable to ART treatment**

| Year | ART coverage | Additional life expectancy (Years) | Multiplier for HIV transmission probability |
| --- | --- | --- | --- |
| 2001 | 0.2 | 0.005 | 0.998 |
| 2002 | 0.5 | 0.014 | 0.995 |
| 2003 | 0.7 | 0.022 | 0.993 |
| 2004 | 1.5 | 0.048 | 0.985 |
| 2005 | 3.3 | 0.105 | 0.967 |
| 2006 | 6.2 | 0.210 | 0.938 |
| 2007 | 9.5 | 0.322 | 0.906 |
| 2008 | 14.4 | 0.487 | 0.857 |
| 2009 | 20.1 | 0.680 | 0.801 |
| 2010 | 25.8 | 0.872 | 0.744 |
| 2011 | 31.5 | 1.065 | 0.688 |
| 2012 | 37.2 | 1.258 | 0.632 |
| 2013 | 42.9 | 1.450 | 0.575 |
| 2014 | 48.6 | 1.643 | 0.519 |
| 2015 | 54.3 | 1.836 | 0.462 |
| 2016 | 60.0 | 2.028 | 0.406 |

**Incorporating microbicide use into the model**

Microbicide was incorporated into the model in a similar way to condom use, as in Foss *et al.* (2003) [16], but dividing the population into two groups: those with access to microbicides (i.e. those covered/reached by the programme of introduction of microbicides into the setting), and those without access to microbicides.

**Calculating the imputed HIV and HSV-2 efficacies of the microbicide**

These per sex-act efficacy estimates are important complements to the trial results, as they are a measure of the intrinsic efficacy of the gel, and provide an indication of how much protection an individual woman could potentially achieve if she used the gel consistently. These imputed efficacy values are also key inputs for models that aim to estimate the public health impact of gel introduction in different settings.

In the CAPRISA 004 trial, participants had different combinations of gel and condom use, including both concurrent and non-current condom and gel use. If condom and gel use are assumed to be independent, and the relative protective efficacy of the gel is assumed to be the same whether or not a condom is used, then the imputed per sex-act HIV and HSV-2 efficacies, and the associated confidence intervals around these estimates, can be estimated by dividing the trial effectiveness (39% (95% CI 6-60%) for HIV and 51% (95% CI 22-70%) for HSV-2) by the percentage of all reported sex-acts (mean 5 sex-acts per month x 24 months x 884 sexually active women followed-up = 106,080 sex-acts) in which gel was used (72% of 106,080 sex-acts) [17-20]. The underlying mathematical rationale behind this calculation can be found in the online supplementary material of Heise *et al.* (2011) [19].

The imputed HIV and HSV-2 efficacies of the gel were 54% (95% CI 8-83%) and 71% (95% CI 30-97%) per sex-act, respectively. These estimates are based upon the trial measures of 72% gel adherence, and the assumption that gel and condom use is independent. The trial measure of gel adherence was estimated using data on the number of gel applicators returned and the number of self-reported sex-acts by trial participants. If the number of applicators returned overestimated the number of gel doses used correctly or if the number of sex-acts was under-reported, then our imputed efficacy estimates will be underestimates. However, applicator returns correlated well with vaginal tenofovir concentration measured among the women involved in a pharmacokinetics sub-study, suggesting that the gel consistency measure is fairly accurate [21]. If we relax the assumption that gel and condom use is independent (and use a more complex equation), then the imputed efficacies are slightly higher than those we presented, suggesting that if anything, our imputed efficacy estimates are underestimates [19, 22]. Moreover, the trial estimate of 72% adherence is based on gel being used within 12 hours both prior to and after sex. If a woman used gel only before or after sex it may have still offered some limited protection, meaning that a higher consistency of use would perhaps be more appropriate to use, leading to lower imputed efficacy estimates. Fortunately, we are reassured of the validity of our imputed efficacy estimates by the report that women in the trial who were ‘high adherers’ (used both doses of the gel in over 80% of sex-acts) reduced their risk of HIV acquisition by 54% (95% CI 4-80%) [17].

**Table S2: Behavioural and demographic parameters used in the model for Gauteng Province.**

| **Model Parameter Definition** | **Parameter uncertainty range** | **Source / Remarks** | **Best Fit Value** |
| --- | --- | --- | --- |
| Population size | 2,918,843 males  2,788,114 females | [23] | 2,918,843 males  2,788,114 females |
| Proportion of the population in each sexual activity group |  | [24-26]  Upper bound of proportion of women who sell sex based on ‘payment for sex’ question in BSS data RHRU 2000^1^ |  |
| *Male no sexual activity* | 0.04 |  | 0.4 |
| *Male main partnerships only* | 0.37-0.55 |  | 0.51 |
| *Male main and casual partnerships* | 0.15-0.33 |  | 0.18 |
| *Male main, casual and commercial partnerships* | 0.22-0.30 |  | 0.28 |
| *Female no sexual activity* | 0.05-0.07 |  | 0.05 |
| *Female main partnerships* | 0.55-0.76 |  | 0.6 |
| *Female main and casual partnerships* | 0-30 |  | 0.32 |
| *Female main, casual and commercial partnerships* | 0.02 – 0.064 |  | 0.03 |
| Mean duration of sex work | 23.5 - 70 months | [25]  Used mean from data as low and doubled the upper CI for upper bound due to uncertainty | 35.6 months |
| Duration of sexual activity | 30 years |  | 30 years |
| Life expectancy | Males 50 years  Females 53 years | [23] | Males 50 years  Females 53 years |
| Rate of new main sex partners |  |  |  |
| *For those engaging in main partnerships only* | 0.167 per month | [24-26] | 0.167 per month |
| *For those engaging in main and casual partnerships* | 0.83 per month |  | 0.83 per month |
| *For those engaging in main, casual and commercial partnerships* | 0.367 per month |  | 0.367 per month |
| Number of sex-acts per FSW with each client on each visit | 1 – 2 | Assume 1-2 due to lack of data from setting | 1.34 |
| Number of clients per sex worker | 32 (26-42) per month | [25, 27] | 26.9 per month |
| Number of casual partners per month |  |  |  |
| *For those engaging in main and casual partnerships* | 1.2 - 1.7 per month | [24-26] | 1.41 per month |
| *For those engaging in main, casual and commercial partnerships* | 0.3 - 1 per month |  | 0.8 per month |
| Number of casual sex-acts per month |  |  |  |
| *For those engaging in main and casual partnerships* | 1.5 – 3 | [24-26] | 2.27 per month |
| *For those engaging in main, casual and commercial partnerships* | 3 – 5 | [24-26] | 4.04 per month |
| Number of main partnership sex-acts for those who have just main, main and casual, and main casual and commercial partnerships |  |  |  |
| *For those engaging in main partnerships only* | 4.6 – 5.7 per month | [24, 26] | 5.7 per month |
| *For those engaging in main and casual partnerships* | 4.2 – 5.2 per month |  | 4.4 per month |
| *For those engaging in main , casual and commercial partnerships* | 8 – 16 per month |  | 10 per month |
| Proportion of commercial sex-acts in which a condom is used | 0.6 – 0.9 | [28] | 0.78 |
| Proportion of sex-acts in casual partnerships in which a condom is used | 0.25 – 0.5 | [24-26] | 0.4 |
| Proportion of sex-acts in main partnerships in which a condom is used | 0.04 – 0.1 | [24-26] | 0.1 |

^1^ The proportion of women who sell sex was fixed between 0.01 and 0.03, and the proportions in the others sexual activity classes were then adjusted (proportionate

to size) either upwards or downwards to ensure that the sum of all sexual activity group proportions summed to 1.

| **Table S3: Biological parameters used in the model for Gauteng Province.** | | | |
| --- | --- | --- | --- |
| **Model Parameter Definition** | **Parameter uncertainty range** | **Source / Remarks** | **Best Fit Value** |
| Duration of initial high viral load phase of HIV | 1.7 (1.64 – 2.5) months | [29-31] | 1.97 months |
| Duration between late stage pre-AIDS heightened viraemia and stopping sex-activity due to AIDS | 12 - 24 months | [29, 32-37, 38 ] | 12.87 months |
| Duration between end of initial viraemia period and start of pre-AIDS heightened viraemia before AIDS | 68 – 74 months | [29-30] | 74.5 months |
| HIV transmission probabilities per sex-act |  |  |  |
| Female to Male | 0.002 (0.0004-0.0015)^1^ | [29-30, 39-44] | 0.0004 |
| Male to Female | 0.01 (0.0004-0.004)^1^ |  | 0.0020 |
| Multiplicative factor for HIV transmission during initial high infectivity phase | 5.5 – 24 | [29-30, 45-46] | 22.5 |
| Multiplicative factor for HIV transmission during pre-AIDS high infectivity phase | 1.9-11.7 | [29-30, 45-46] | 3.5 |
| Duration of *Treponema pallidum* (syphilis) primary phase in males and females | 6 (2 – 8) months | [9, 47] | 3.7 months |
| Duration of *Treponema pallidum* (syphilis) primary phase in FSW | 6 (2 – 8) months | [9, 47] | 6.7 months |
| Duration of *Treponema pallidum* (syphilis) secondary phase | 4 – 6 months | [9, 47] | 4.6 months |
| Duration of *Treponema pallidum* (syphilis) latent phase | 3 – 24 months | [9, 47] | 5.4 months |
| Duration of *Treponema pallidum* (syphilis) immune phase | 1 – 60 months | [9, 47] | 36.6 months |
| *Treponema pallidum* (syphilis) transmission probability per sex-act | 0.3 (0.1-0.6) | [9, 29, 48-51] | 0.274 |
| *Treponema pallidum* (syphilis) multiplicative cofactor effect per sex-act for increasing HIV transmission | 1.3 – 4.4 | [29, 48, 52-53] | 1.59 |
| Duration of bacterial STIs *(Trichomonas vaginalis, Chlamydial Trachomtis and Neisseria Gonorhoea)* | 2-5 months | [54] [55] | 2.12 months |
| Duration of bacterial STIs in FSW | 0.5- 3months | [54] [55] | 1.68 months |
| Bacterial STI transmission probability per sex-act | 0.05-0.2 | [56-60] | 0.16 |
| Bacterial STI multiplicative cofactor effect per sex-act for increasing HIV transmission | 1.2 - 6 | [48, 52-53] | 2.95 |
| Duration of primary HSV2 | 0.36 – 0.66 months | [61-66] | 0.6 months |
| Duration of a symptomatic recurrence | 0.01 – 0.16 per month | [61-66] | 0.14 months |
| HSV-2 transmission probability |  |  |  |
| *Primary* | 0.004 – 0.15^2^ | [67-70] | 0.126 |
| *Asymptomatic* | 0.0002 – 0.004^2^ | [67-72] |  |
| *Recurrent* | 0.005 – 0.105^2^ | [67-70] | 0.0045 |
| Multiplicative cofactors for increasing a dually HIV and herpes simplex virus type-2 (HSV-2) infected’s infectivity of HIV |  |  |  |
| *HSV-2-symptomatic* | 1 – 6^3^ | [43, 45, 52, 73-83] | 2.4 |
| *HSV-2-asymptomatic* | 1.05 – 15^3^  (incorporates increased rate of shedding) | [43, 45, 52, 73-83] | 4.1 |
| Multiplicative cofactors for increasing a HIV-negative/HSV-2 infected’s susceptibility to HIV |  |  |  |
| *HSV-2-symptomatic* | 1.5 -9^4^ | [43, 45, 52, 73-83] | 2.96 |
| *HSV-2-asymptomatic* | 1.2 - 6^4^ | [43, 45, 52, 73-83] | 1.89 |
| Monthly rate at which have a symptomatic HSV2 recurrence if HIV-negative | 0.17 (0.08-0.417) ^5^ | [7, 61, 63-65, 84-85] | 0.25 per month |
| Monthly rate at which have a symptomatic recurrence if HIV-positive | 0.25 (0.08 – 1.16) ^5^ | [7, 61, 63-65, 84-85] | 0.49 per month |
| Multiplicative cofactor for increased infectivity of HSV-2 infected person if they also have HIV (incorporating increased asymptomatic shedding among coinfecteds) |  |  |  |
| *Primary* | 1.5– 2.5 | [2, 73, 86-89] | 2.4 |
| *Asymptomatic* | 2 – 4 | [2, 43, 45, 52, 73-74, 76-77, 80-83, 90-91] | 2.7 |
| Multiplicative cofactor for increased susceptibility to HSV2 if HIV infected | 1 – 5 | [45, 79, 82, 92] | 4.9 |
| Condom efficacy (proportion of sex-acts, in which a condom is used, that are protected) | 0.8 – 0.95 | [93-94] | 0.87 |

^1^ Starting with these initial parameter ranges, 30 million parameter sets were generated but no fits were identified. The ranges were reduced iteratively to hone in on the lower half of the ranges since these were producing the most near fits (0.0004 – 0.0007 for females to male and 0.0004 – 0.002 for males to females).

^2^ Starting with these initial parameter ranges, 30 million parameter sets were generated but no fits were identified. The ranges were reduced iteratively to hone in on the part of the ranges producing the most near fits (0.08-0.15 for primary stage, 0.003 – 0.004 for asymptomatic stage and 0.008 – 0.105 for symptomatic stage).

^3^ Starting with these initial parameter ranges, 30 million parameter sets were generated but no fits were identified. The ranges were reduced iteratively to hone in on the part of the ranges producing the most near fits (1-3 for primary stage and 4-10 for asymptomatic stage).

^4^ Starting with these initial parameter ranges, 30 million parameter sets were generated but no fits were identified. The ranges were reduced iteratively to hone in on the part of the ranges producing the most near fits (1.5-5 for primary stage and 1.2-3 for asymptomatic stage).

^5^ Starting with these initial parameter ranges, 30 million parameter sets were generated but no fits were identified. The ranges were reduced iteratively to hone in on the part of the ranges producing the most near fits (0.2 – 0.417 for HIV-negatives and 0.5 – 1.16 for HIV-positives).

The fitting procedure used Latin Hypercube Sampling, with the reduced ranges in footnotes 1-5 above, to generate 2100 behavioural parameter sets and 600 STI parameter sets. These parameter sets resulted in 2008 fits. The 2008 fits were then combined with 3000 HIV/HSV parameter sets (using reduced ranges in footnotes 1-5 above), which produced 134 final model fits. In effect, this resulted in searching over a total of 3.78x10^9^ Latin Hypercube samples.

| **Table S4. Data used in model fitting: Prevalence of Sexually Transmitted Infections – Gauteng / South Africa** | | | | |
| --- | --- | --- | --- | --- |
| **STI** | **Group** | **Prevalence Data** | | **Value from**  **best-fit model** |
|  |  | **Range used in fitting** | **Source** |  |
| **HIV** | *General population males* | 12.8% (11.5 – 15.5%) [2002] | [95-96] | 12% |
|  | *General population females* | 17.7 % (15.4 – 20.7%) [2002] |  | 18% |
|  | *General population males* | 11.2 % (8.5 – 14%) [2005] |  | 12% |
|  | *General population females* | 21.1% (17.5 – 25%) [2005] |  | 19% |
|  | *General population (all)* | 16.9% (15.5 – 18.4%) [2008] | [97] | 16% |
|  | *Female sex workers (FSW)* | 40 – 67% [1997] | [28, 98] | 51% |
|  | *Clients* | <42.5% [2000] | [99] | 14% |
| Bacterial STIs  *(Chlamydial Trachomtis and Neisseria Gonorhoea)* | *FSW* | 17% – 55% [1997] | [100] | 29% |
| *Treponema pallidum* (syphilis) | *FSW* | 25% (20 -31%) [1997] | [28] | 28% |
|  | *Client* | <45% | No Data | 10% |
| Herpes simplex virus type-2 | *General population females* | 58% (51.35-72.5% ) | [17, 97] | 55% |

**Table S5 Cost inputs, assumptions and sources.**

| **Provider cost inputs** | **Cost estimation (All presented in 2012 US$)** | **Source** |
| --- | --- | --- |
| Product costs   - Low - High   Quantity: | $0.17  $0.56  1 and 2 doses per sex-act plus 10% wastage, accounting for different use consistency as determined by scenario. | Expert opinion on possible range when produced at scale. [101] |
| Training costs | $1,535 per health facility  Rolled out over 3 years, with a short refresher training every 3 years at 1/3 of the intensity (and cost) per clinic. | [102] |
| Facility delivery costs | Initiation visit (once per new woman reached): 20 minutes with enrolled nurse ($7.36 per hour direct cost).  Annual adherence counselling and gel collection visits (ranging from 2, 4, 6 per year): 10 minutes with an auxiliary nurse ($5.80).  With a mark-up of 100% to account for other facility costs.  Salary costs based on public-sector salary data, assuming 46 working weeks per year and a 40 hour work week. | Salary costs [103]  Visit durations [104]  Mark-up for indirect costs {cleary 2004 Table 9. |
| HIV testing  Cost  Frequency per year | $9.52 per round of testing.  2, 3, 4, 6 | [105]  Expert opinion on reasonable range. |
| Mass media campaign | $1,041,655 in years 1-5, 8, 11, 14. | [106] |
| **Effectiveness inputs** |  |  |
| Age at HIV infection | 26.6 | [107] |
| Life expectancies from date of HIV infection No ART  With ART | 10 years, of which 30% asymptomatic pre AIDS, 50% symptomatic pre AIDS, and 20% with AIDS  Near full life expectancy: 34 from age of infection years of which 2.59 years are asymptomatic pre AIDS, 0.41 year is symptomatic pre AIDS, 31 years on of AIDS on ART, | [108]  [109-110] [111-112] |
| Disability weights  Pre AIDS asymptomatic  HIV cases: symptomatic, pre-AIDS  HIV/AIDS cases: receiving antiretroviral treatment  AIDS cases: not receiving antiretroviral treatment | 0.051  0.221  0.053  0.547 | [113]  [114] |
| DALYs averted by preventing an HIV infection  No ART  ART (near full life expectancy) | 21.84  5.99  No further changes were made to the standard DALY equation as presented in Fox-Rushby et al. (2001) | [115], [116] |
| **HIV treatment cost inputs** | | |
| Access to ART | 52% of people eligible for ART ( at a CD4 <350), with 80% in sensitivity analysis | [117]{South African Department of Health, 2012 #196} |
| Duration on  1^st^ line ART  2^nd^ line ART | (during year 1-3 post infection, the patient is not yet eligible for ART)  in year 4-16 post infection  in year 17-34 post infection  Note: Lodi (2011) shows that with HIV subtype C, the most common type in Southern Africa, the medium duration to reach cd4 count below 350 is 2.59 year, however, taking into account that people may not immediately be identified or initiated, 3 years was chosen as the period of no ART. | [118],[119] (for South Africa Subtype). |
| Annual ART costs 1^st^ line  2^nd^ line | USD 576 for 13 years  USD 1114 for 15 years  Note: These are the adult average annual costs of the 2012 South African Treatment guidelines, which include the full cost of delivering 1^st^ line ART for the first 13 months, and thereafter 2^nd^ line ART. | [120-121] |
| If no ART, lifetime health care costs | $3900, of which 50% in last year (year 10) | [122] |
| General inputs |  |  |
| Discount rate | Central: 3%  Upper: 6%  Lower: 0% |  |

**References**

1. Vickerman P, Watts C, Delany S, Alary M, Rees H, Heise L: **The importance of context: model projections on how microbicide impact could be affected by the underlying epidemiologic and behavioral situation in 2 African settings.** *Sex Transm Dis* 2006, **33:**397-405.

2. Vickerman P, Foss AM, Pickles M, Deering K, Verma S, Demers E, Moses S, Alary M, Boily M-C: **Is the Indian HIV epidemic driven by commercial sex? A modelling analysis from south India.** *AIDS* 2010, **24:**2563-2572.

3. Garnett GP, Anderson RM: **Strategies for limiting the spread of HIV in developing countries: conclusions based on studies of the transmission dynamics of the virus.** *J Acquir Immune Defic Syndr Hum Retrovirol* 1995, **9:**500-513.

4. Rehle TM, Saidel TJ, Hassig SE, Bouey PD, Gaillard EM, Sokal DC: **AVERT: a user-friendly model to estimate the impact of HIV/sexually transmitted disease prevention interventions on HIV transmission.** *AIDS* 1998, **12 Suppl 2:**S27-35.

5. Weinstein MC, Graham JD, Siegel JE, Fineberg HV: **Cost-effectiveness analysis of AIDS prevention programs: concepts, complications and illustrations.** In *Confronting AIDS.* Edited by Turner CF, Miller HG, Moses LE. Washington DC: National Academy Press; 1989: 471-499

6. Foss AM, Vickerman PT, Chalabi Z, Mayaud P, Alary M, Watts C: **Dynamic modelling of herpes simplex virus type 2 (HSV-2) transmission: issues in structural uncertainty.** *Bulletin of Mathematical Biology* 2009, **71:**720-749

7. Foss AM, Vickerman PT, Mayaud P, Weiss H, Ramesh BM, Reza-Paul S, Washington R, Blanchard J, Moses S, Lowndes CM, et al: **Modelling the interactions between HSV-2 and HIV: implications for the HIV epidemic in southern India.** *Sex Transm Infect* 2011, **87:**22-27.

8. Grassly NC, Fraser C, Garnett GP: **Host immunity and synchronized epidemics of syphilis across the United States.** *Nature* 2005, **433:**417-421.

9. Garnett GP, Aral SO, Hoyle DV, Cates W, Jr., Anderson RM: **The natural history of syphilis. Implications for the transmission dynamics and control of infection.** *Sex Transm Dis* 1997, **24:**185-200.

10. Pourbohloul B, Rekart ML, Brunham RC: **Impact of mass treatment on syphilis transmission: a mathematical modeling approach.** *Sex Transm Dis* 2003, **30:**297-305.

11. Adam MA, Johnson LF: **Estimation of adult antiretroviral treatment coverage in South Africa.** *S Afr Med J* 2009, **99:**661-667.

12. Cohen M, Chen Y, McCauley M, Gamble T, Hosseinipour M, Kumarasamy N, al. e: **Prevention of HIV-1 infection with early antiretroviral therapy.** *N Engl J Med* 2011, **365:**493-505.

13. Cox A, Foss A, Shafer L-A, Nsubuga R, Vickerman P, Hayes R, Watts C, White R: **Attaining realistic and significant reductions in HIV Incidence: Combining microbicide and male circumcision interventions in Rural Uganda.** *Sex Transm Infect* 2011, **87:**635-639.

14. **Statistics South Africa.** [ <http://www.statssa.gov.za/> ]

15. Bor J, Herbst A, Newell M, Bärnighausen T: **Increases in adult life expectancy in rural South Africa: valuing the scale-up of**

**HIV treatment.** *Science* 2013, **339:**961-965.

16. Foss A, Vickerman P, Heise L, Watts CH: **Shifts in condom use following microbicide introduction: should we be concerned?** *AIDS* 2003, **17:**1227-1237.

17. Karim QA, Karim SS, Frohlich JA, Grobler AC, Baxter C, Mansoor LE, Kharsany AB, Sibeko S, Mlisana KP, Omar Z, et al: **Effectiveness and Safety of Tenofovir Gel, an Antiretroviral Microbicide, for the Prevention of HIV Infection in Women.** *Science* 2010, **Epub:**<http://www.sciencemag.org/cgi/content/abstract/science.1193748v1193741>.

18. Karim SSA: **Results of the CAPRISA 004 trial of tenofovir gel.** In *XVIII International AIDS Conference 18-23 July; Vienna, Austria*. 2010

19. Heise L, Watts C, Foss A, Vickerman P, Trussell J, Hayes R, McCormack S: **Apples and oranges? Interpreting success in HIV prevention trials.** *Contraception* 2011, **83:**10-15.

20. Vickerman P, Foss A, Watts C: **Using modeling to explore the degree to which a microbicide's sexually transmitted infection efficacy may contribute to the HIV effectiveness measured in phase 3 microbicide trials.** *J Acquir Immune Defic Syndr* 2008, **48:**460-467.

21. Kashuba AD, Abdool Karim SS, Kraft E, White N, Sibeko S, Werner L, Mansoor LE, Gengiah T, Sidhoo S, Abdool Karim Q: **Do systemic and genital tract tenofovir concentrations predict HIV seroconversion in the CAPRISA 004 tenofovir gel trial?** In *XVIII International AIDS Conference 18-23 July; Vienna, Austria*. 2010

22. Trussell J, Dominik R: **Will microbicide trials yield unbiased estimates of microbicide efficacy?** *Contraception* 2005, **72:**408-413.

23. **Statistics South Africa.** [ <http://www.statssa.gov.za/> ]

24. RHRU: **Reproductive Health Research Unit Behavioural Sentinel Survey data report - Clients of family planning - Gauteng.**; 2000

25. RHRU: **Reproductive Health Research Unit Behavioural Sentinel Survey data report - Commercial sex workers - Gauteng.**; 2000

26. RHRU: **Reproductive Health Research Unit Behavioural Sentinel Survey data report - Male STD clinic attenders - Gauteng.**; 2000

27. Delany S: **Summary of behavioural and epidemiological data from Hillbrow sex worker intervention.** Reproductive Health Research Unit; 2001

28. Dunkle K, Beksinka M, Rees H, Ballard R, Htun Y, Wilson ML: **Risk factors for HIV infection among sex workers in Johannesburg, South Africa.** *Int J STD AIDS* 2005, **16:**256 - 261.

29. White RG, Orroth KK, Korenromp EL, Bakker R, Wambura M, Sewankambo NK, Gray RH, Kamali A, Whitworth JA, Grosskurth H, et al: **Can population differences explain the contrasting results of the Mwanza, Rakai, and Masaka HIV/sexually transmitted disease intervention trials?: a modeling study.** *J Acquir Immune Defic Syndr* 2004, **37:**1500-1513.

30. Wawer MJ, Gray RH, Sewankambo NK, Serwadda D, Li X, Laeyendecker O, Kiwanuka N, Kigozi G, Kiddugavu M, Lutalo T, et al: **Rates of HIV-1 Transmission per Coital Act, by Stage of HIV-1 Infection, in Rakai, Uganda.** *J Infect Dis* 2005, **191:**1403-1409.

31. Pilcher CD, Tien H, Vernazza PL, Stewart P, Chakraborty H, Eron JJ, Jr., Cohen MS: **Semen viral dynamics in acute HIV infection: implications for sexual transmission [Abstract ThOrC1489].** In *14th International AIDS Conference; Barcelona, Spain*. 2002

32. Srinivasa Rao AS, Hira SK: **Evidence of shorter incubation period of HIV-1 in Mumbai, India.** *Int J STD AIDS* 2003, **14:**499-500.

33. Hira SK, Shroff HJ, Lanjewar DN, Dholkia YN, Bhatia VP, Dupont HL: **The natural history of human immunodeficiency virus infection among adults in Mumbai.** *Natl Med J India* 2003, **16:**126-131.

34. Ruxrungtham K, Phanuphak P: **Update on HIV/AIDS in Thailand.** *J Med Assoc Thai* 2001, **84 Suppl 1:**S1-17.

35. Floridia M, Fragola V, Galluzzo CM, Giannini G, Pirillo MF, Andreotti M, Tomino C, Vella S: **HIV-related morbidity and mortality in patients starting protease inhibitors in very advanced HIV disease (CD4 count of < 50 cells/microL): an analysis of 338 clinical events from a randomized clinical trial.** *HIV Med* 2002, **3:**75-84.

36. Over M, Piot P: **Human immunodeficiency virus infection and other sexually transmitted diseases in developing countries: public health importance and priorities for resource allocation.** *J Infect Dis* 1996, **174 Suppl 2:**S162-175.

37. Post FA, Badri M, Wood R, Maartens G: **AIDS in Africa--survival according to AIDS-defining illness.** *S Afr Med J* 2001, **91:**583-586.

38. Morgan D, Mahe C, Mayanja B, Whitworth JAG: **Progression to symptomatic disease in people infected with HIV-1 in rural Uganda: prospective cohort study.** *BMJ* 2002, **324:**193-196.

39. Royce RA, Sena A, Cates W, Jr., Cohen MS: **Sexual transmission of HIV.** *N Engl J Med* 1997, **336:**1072-1078.

40. Mastro TD, de Vincenzi I: **Probabilities of sexual HIV-1 transmission.** *AIDS* 1996, **10 Suppl A:**S75-82.

41. Leynaert B, Downs AM, de Vincenzi I: **Heterosexual transmission of human immunodeficiency virus: variability of infectivity throughout the course of infection. European Study Group on Heterosexual Transmission of HIV.** *Am J Epidemiol* 1998, **148:**88-96.

42. Korenromp EL, Van Vliet C, Grosskurth H, Gavyole A, Van der Ploeg CP, Fransen L, Hayes RJ, Habbema JD: **Model-based evaluation of single-round mass treatment of sexually transmitted diseases for HIV control in a rural African population.** *AIDS* 2000, **14:**573-593.

43. Gray RH, Wawer MJ, Brookmeyer R, Sewankambo N, Serwadda D, Wabwire Mangen F, Lutalo T, Li X, van Cott T, Quinn TC: **probability of HIV-1 transmission per coital act in monogamous, heterosexual, HIV-1-discordant couples in Rakai, Uganda.** *Lancet* 2001, **357:**1149-1153.

44. Baggaley R, Boily MC, White RG, Alary M: **Systematic review of HIV-1 transmission probabilities in absence of antiretroviral therapy.** London: Imperial College; 2004

45. Quinn TC, Wawer MJ, Sewankambo N, Serwadda D, Li C, Wabwire Mangen F, Meehan MO, Lutalo T, Gray RH: **Viral load and heterosexual transmission of human immunodeficiency virus type 1. Rakai Project Study Group.** *N Engl J Med* 2000, **342:**921-929.

46. Pilcher CD, Tien HC, Eron JJ, Jr., Vernazza PL, Leu SY, Stewart PW, Goh LE, Cohen MS: **Brief but efficient: acute HIV infection and the sexual transmission of HIV.** *J Infect Dis* 2004, **189:**1785-1792.

47. Sparling PF: **Chapter 34: Natural history of syphilis.** In *Sexually Transmitted Diseases.* Third edition. Edited by Holmes KK, Mardh P, Sparling PF, Lemon SM, Stamm WE, Piot P, Wasserheit JN. USA: McGraw-Hill; 1999: 473-478

48. Sexton J, Garnett G, Rottingen JA: **Metaanalysis and metaregression in interpreting study variability in the impact of sexually transmitted diseases on susceptibility to HIV infection.** *Sex Transm Dis* 2005, **32:**351-357.

49. Schroeter AL, Turner RH, Lucas JB, Brown WJ: **Therapy for incubating syphilis. Effectiveness of gonorrhea treatment.** *Jama* 1971, **218:**711-713.

50. Cates W, Jr., Rothenberg RB, Blount JH: **Syphilis control. The historic context and epidemiologic basis for interrupting sexual transmission of Treponema pallidum.** *Sex Transm Dis* 1996, **23:**68-75.

51. Hook EW, 3rd: **Biomedical issues in syphilis control.** *Sex Transm Dis* 1996, **23:**5-8.

52. Rottingen JA, Cameron DW, Garnett GP: **A systematic review of the epidemiologic interactions between classic sexually transmitted diseases and HIV: how much really is known?** *Sex Transm Dis* 2001, **28:**579-597.

53. Laga M, Manoka A, Kivuvu M, Malele B, Tuliza M, Nzila N, Goeman J, Behets F, Batter V, Alary M, et al.: **Non-ulcerative sexually transmitted diseases as risk factors for HIV-1 transmission in women: results from a cohort study.** *AIDS* 1993, **7:**95-102.

54. Korenromp EL, Sudaryo MK, de Vlas SJ, Gray RH, Sewankambo NK, Serwadda D, Wawer MJ, Habbema JD: **What proportion of episodes of gonorrhoea and chlamydia becomes symptomatic?** *Int J STD AIDS* 2002, **13:**91-101.

55. Golden MR, Schillinger JA, Markowitz L, St Louis ME: **Duration of untreated genital infections with chlamydia trachomatis: a review of the literature.** *Sex Transm Dis* 2000, **27:**329-337.

56. Holmes KK, Johnson DW, Trostle HJ: **An estimate of the risk of men acquiring gonorrhea by sexual contact with infected females.** *Am J Epidemiol* 1970, **91:**170-174.

57. Lycke E, Lowhagen GB, Hallhagen G, Johannisson G, Ramstedt K: **The risk of transmission of genital Chlamydia trachomatis infection is less than that of genital Neisseria gonorrhoeae infection.** *Sex Transm Dis* 1980, **7:**6-10.

58. Hooper RR, Reynolds GH, Jones OG, Zaidi AA, Wiesner PJ, Latimer KP: **Cohort study of venereal disease. 1: The risk of gonorrhoea transmission from infected women to men.** *American journal of Epidemiology* 1978, **108:**136-144.

59. Ruijs GJ, Schut IK, Schirm J, Schroder FP: **Prevalence, incidence, and risk of acquiring urogenital gonococcal or chlamydial infection in prostitutes working in brothels.** *Genitourinary Medicine* 1988, **64:**49-51.

60. Quinn SC, Gaydos C, Shepherd ME, Bobo L, Hook EW, 3rd, Viscidi R, Rompalo A: **Epidemiologic and microbiologic correlates of Chlamydia trachomatis infection in sexual partnerships.** *JAMA* 1996, **276:**1737-1742.

61. Benedetti JK, Zeh J, Corey L: **Clinical reactivation of genital herpes simplex virus infection decreases in frequency over time.** *Ann Intern Med* 1999, **131:**14-20.

62. Cheong WK, Thirumoorthy T, Doraisingham S, Ling AE: **Clinical and laboratory study of first episode genital herpes in Singapore.** *International Journal of STD & AIDS* 1990, **1:**195-198.

63. Corey L, Adams HG, Brown ZA, Holmes KK: **Genital herpes simplex virus infections: clinical manifestations, course, and complications.** *Ann Intern Med* 1983, **98:**958-972.

64. Corey L, Wald A: **Chapter 21: Genital herpes.** In *Sexually Transmitted Diseases.* Third edition. Edited by Holmes KK, Mardh P, Sparling PF, Lemon SM, Stamm WE, Piot P, Wasserheit JN. USA: McGraw-Hill; 1999: 285-312

65. Diamond C, Selke S, Ashley R, Benedetti J, Corey L: **Clinical course of patients with serologic evidence of recurrent genital herpes presenting with signs and symptoms of first episode disease.** *Sex Transm Dis* 1999, **26:**221-225.

66. Koelle DM, Benedetti J, Langenberg A, Corey L: **Asymptomatic reactivation of herpes simplex virus in women after the first episode of genital herpes.** *Ann Intern Med* 1992, **116:**433-437.

67. Bryson Y, Dillon M, Bernstein DI, Radolf J, Zakowski P, Garratty E: **Risk of acquisition of genital herpes simplex virus type 2 in sex partners of persons with genital herpes: a prospective couple study.** *J Infect Dis* 1993, **167:**942-946.

68. Corey L, Wald A, Patel R, Sacks SL, Tyring SK, Warren T, Douglas JM, Jr., Paavonen J, Morrow RA, Beutner KR, et al: **Once-daily valacyclovir to reduce the risk of transmission of genital herpes.** *N Engl J Med* 2004, **350:**11-20.

69. Mertz GJ, Coombs RW, Ashley R, Jourden J, Remington M, Winter C, Fahnlander A, Guinan M, Ducey H, Corey L: **Transmission of genital herpes in couples with one symptomatic and one asymptomatic partner: a prospective study.** *J Infect Dis* 1988, **157:**1169-1177.

70. Wald A, Langenberg AG, Link K, Izu AE, Ashley R, Warren T, Tyring S, Douglas JM, Jr., Corey L: **Effect of condoms on reducing the transmission of herpes simplex virus type 2 from men to women.** *Jama* 2001, **285:**3100-3106.

71. Mahiane SG, Legeai C, Taljaard D, Latouche A, Puren A, Peillon A, Bretagnolle J, Lissouba P, Nguema EP, Gassiat E, Auvert B: **Transmission probabilities of HIV and herpes simplex virus type 2, effect of male circumcision and interaction: a longitudinal study in a township of South Africa.** *AIDS* 2009, **23:**377-383.

72. Wald A, Langenberg AGM, Krantz E, Douglas JM, Jr., Handsfield HH, DiCarlo RP, Adimora AA, Izu AE, Morrow RA, Corey L: **The Relationship between Condom Use and Herpes Simplex Virus Acquisition.** *Ann Intern Med* 2005, **143:**707-713.

73. Corey L, Wald A, Celum CL, Quinn TC: **The Effects of Herpes Simplex Virus-2 on HIV-1 Acquisition and Transmission: A Review of Two Overlapping Epidemics.** *J Acquir Immune Defic Syndr* 2004, **35:**435-445.

74. Reynolds SJ, Risbud AR, Shepherd ME, Zenilman JM, Brookmeyer RS, Paranjape RS, Divekar AD, Gangakhedkar RR, Ghate MV, Bollinger RC, Mehendale SM: **Recent herpes simplex virus type 2 infection and the risk of human immunodeficiency virus type 1 acquisition in India.** *J Infect Dis* 2003, **187:**1513-1521.

75. Korenromp EL, Sake Jdv, Nagelkerke N, Habbema JD: **Estimating the magnitude of STD cofactor effects on HIV transmission.** *Sex Transm Dis* 2001, **28:**613-621.

76. Wald A, Link K: **Risk of human immunodeficiency virus infection in herpes simplex virus type 2-seropositive persons: a meta-analysis.** *J Infect Dis* 2002, **185:**45-52.

77. Ramjee G, Williams B, Gouws E, Van Dyck E, Deken BD, Karim SA: **The Impact of Incident and Prevalent Herpes Simplex Virus-2 Infection on the Incidence of HIV-1 Infection Among Commercial Sex Workers in South Africa.** *J Acquir Immune Defic Syndr* 2005, **39:**333-339.

78. Freeman EE, Weiss HA, Glynn JR, Cross PL, Whitworth JA, Hayes RJ: **Herpes simplex virus 2 infection increases HIV acquisition in men and women: systematic review and meta-analysis of longitudinal studies.** *AIDS* 2006, **20:**73-83.

79. Mbopi-Keou FX, Gresenguet G, Mayaud P, Weiss HA, Gopal R, Matta M, Paul JL, Brown DW, Hayes RJ, Mabey DC, Belec L: **Interactions between herpes simplex virus type 2 and human immunodeficiency virus type 1 infection in African women: opportunities for intervention.** *J Infect Dis* 2000, **182:**1090-1096.

80. Schacker T, Zeh J, Hu H, Shaughnessy M, Corey L: **Changes in plasma human immunodeficiency virus type 1 RNA associated with herpes simplex virus reactivation and suppression.** *J Infect Dis* 2002, **186:**1718-1725.

81. Wright PW, Hoesley CJ, Squires KE, Croom-Rivers A, Weiss HL, Gnann JW, Jr.: **A prospective study of genital herpes simplex virus type 2 infection in human immunodeficiency virus type 1 (HIV-1)-seropositive women: correlations with CD4 cell count and plasma HIV-1 RNA level.** *Clin Infect Dis* 2003, **36:**207-211.

82. Serwadda D, Gray RH, Sewankambo NK, Wabwire-Mangen F, Chen MZ, Quinn TC, Lutalo T, Kiwanuka N, Kigozi G, Nalugoda F, et al: **Human immunodeficiency virus acquisition associated with genital ulcer disease and herpes simplex virus type 2 infection: a nested case-control study in Rakai, Uganda.** *J Infect Dis* 2003, **188:**1492-1497.

83. Mole L, Ripich S, Margolis D, Holodniy M: **The impact of active herpes simplex virus infection on human immunodeficiency virus load.** *J Infect Dis* 1997, **176:**766-770.

84. Benedetti J, Corey L, Ashley R: **Recurrence rates in genital herpes after symptomatic first-episode infection.** *Ann Intern Med* 1994, **121:**847-854.

85. Langenberg AG, Corey L, Ashley RL, Leong WP, Straus SE: **A prospective study of new infections with herpes simplex virus type 1 and type 2. Chiron HSV Vaccine Study Group.** *N Engl J Med* 1999, **341:**1432-1438.

86. Schacker T, Zeh J, Hu HL, Hill E, Corey L: **Frequency of symptomatic and asymptomatic herpes simplex virus type 2 reactivations among human immunodeficiency virus-infected men.** *J Infect Dis* 1998, **178:**1616-1622.

87. Nagot N, Foulongne V, Becquart P, Mayaud P, Konate I, Ouedraogo A, al. e: **Longitudinal assessment of HIV-1 and HSV-2 shedding in the genital tract of West African women.** *J Acquir Immune Defic Syndr* 2005, **39:**632-634

88. Augenbraun M, Feldman J, Chirgwin K, Zenilman J, Clarke L, DeHovitz J, Landesman S, Minkoff H: **Increased Genital Shedding of Herpes Simplex Virus Type 2 in HIV-Seropositive Women.** *Ann Intern Med* 1995, **123:**845-847.

89. LeGoff J, Weiss HA, Gresenguet G, Nzambi K, Frost E, Hayes RJ, al. e: **Cervicovaginal HIV-1 and herpes simplex virus type 2 shedding during genital ulcer disease episodes.** *AIDS* 2007, **21:**1569-1578.

90. Hennessey KA, Giorgi JV, Kaplan AH, Visscher BR, Gange S, Margolick JB, Riddler S, Phair J, Detels R: **AIDS onset at high CD4+ cell levels is associated with high HIV load.** *AIDS Res Hum Retroviruses* 2000, **16:**103-107.

91. McClelland RS, Wang CC, Overbaugh J, Richardson BA, Corey L, Ashley RL, Mandaliya K, Ndinya-Achola J, Bwayo JJ, Kreiss JK: **Association between cervical shedding of herpes simplex virus and HIV-1.** *AIDS* 2002, **16:**2425-2430.

92. Wald A, Huang ML, Carrell D, Selke S, Corey L: **Polymerase chain reaction for detection of herpes simplex virus (HSV) DNA on mucosal surfaces: comparison with HSV isolation in cell culture.** *J Infect Dis* 2003, **188:**1345-1351.

93. Weller S, Davis K, Woolthuis EP: **Condom effectiveness in reducing heterosexual HIV transmission (review).** Oxford: The Cochrane Database of Systematic Reviews 2002, Issue 1. Art. No.: CD003255. DOI: 10.1002/14651858.CD003255. ; 2002 [Updated 2005]

94. Pinkerton SD, Abramson PR: **Effectiveness of condoms in preventing HIV transmission.** *Social Science and Medicine* 1997, **44:**1303-1312.

95. Shisana O, Rehle T, Simbayi L, Parker W, Zuma K, Bhana A, Connolly C, Jooste S, Pillay V: **South African National HIV Prevalence, HIV Incidence, Behaviour and Communication Survey.**; 2005

96. Shisana O, Simbayi L: **Nelson Mandela/HSRC study of HIV/AIDS. South African National HIV prevalence, behavioural risks and mass media.** Cape Town: The human sciences research council; 2002

97. Shisana O, Rehle T, Simbayi L, Zuma K, Jooste S, Pillay-van-Wyk V, Mbelle N, Van Zyl J, Parker W, Zungu N, et al: **South African national HIV prevalence, incidence, behaviour and communication survey. A turning tide among teenagers? .** Cape Town: HSRC Press; 2009

98. Ramjee G, Karim SS, Sturm AW: **Sexually transmitted infections among sex workers in KwaZulu-Natal, South Africa.** *Sex Transm Dis* 1998, **25:**346-349.

99. Ballard, al e: **HIV / RPR surveillance report among STD clinic attenders. South Africacn Institute of Medical Research.** 2000.

100. Johnson LF, Coetzee DJ, Dorrington RE: **Sentinel surveillance of sexually transmitted infections in South Africa: a review.** *Sex Transm Infect* 2005, **81:**287-293.

101. Friend D: **Tenofovir 1% gel, current prices and expected prices at different scales.** In *Book Tenofovir 1% gel, current prices and expected prices at different scales*. City; 2012.

102. Terris-Prestholt F, Kumaranayake L, Obasi AI, Cleophas-Mazige B, Makokha M, Todd J, Ross DA, Hayes RJ: **From trial intervention to scale-up: costs of an adolescent sexual health program in Mwanza, Tanzania.** *Sex Transm Dis* 2006, **33:**S133-139.

103. Meyer-Rath G: **Public sector salary costs.** In *Book Public sector salary costs*. City; 2009.

104. Terris-Prestholt F, Michaels C, Kumaranayake L, Meyer-Rath G, Watts C: **Costing Microbicide Distribution: Estimating cost-effectiveness & modelling potential role out.** In *Book Costing Microbicide Distribution: Estimating cost-effectiveness & modelling potential role out*. City; 2009.

105. Bassett IV, Giddy J, Nkera J, Wang B, Losina E, Lu Z, Freedberg KA, Walensky RP: **Routine voluntary HIV testing in Durban, South Africa: the experience from an outpatient department.** *J Acquir Immune Defic Syndr* 2007, **46:**181-186.

106. Bollinger L, Stover, J., Boulle, A., Cleary, S. : **Database of Unit Costs for HIV and AIDS interventions in South Africa: Resource Needs for HIV/AIDS: Model for estimating resources needed for prevention, care and mitigation.** In *Book Database of Unit Costs for HIV and AIDS interventions in South Africa: Resource Needs for HIV/AIDS: Model for estimating resources needed for prevention, care and mitigation.* City: Centre for Economic Governance and AIDS in Africa.; 2006.

107. Vickerman P, Terris-Prestholt F, Delany S, Kumaranayake L, Rees H, Watts C: **Are targeted HIV prevention activities cost-effective in high prevalence settings? Results from a sexually transmitted infection treatment project for sex workers in Johannesburg, South Africa.** *Sex Transm Dis* 2006, **33:**S122-132.

108. Murray C, Lopez A: **The Global Burden of Disease.** In *Book The Global Burden of Disease*. City: Harvard University Press.; 1996.

109. Johnson LF, Mossong J, Dorrington RE, Schomaker M, Hoffmann CJ, Keiser O, Fox MP, Wood R, Prozesky H, Giddy J, et al: **Life expectancies of South African adults starting antiretroviral treatment: collaborative analysis of cohort studies.** *PLoS Med* 2013, **10:**e1001418.

110. Verguet S, Walsh JA: **Further benefits by early start of HIV treatment in South Africa and the USA.** *Sex Transm Infect* 2010, **7**.

111. Johansson KA, Robberstad B, Norheim OF: **Further benefits by early start of HIV treatment in low income countries: Survival estimates of early versus deferred antiretroviral therapy.** *AIDS research and therapy* 2010, **7**.

112. Mills EJ, Bakanda C, Birungi J, Chan K, Ford N, Cooper CL, Nachega JB, Dybul M, Hogg RS: **Life expectancy of persons receiving combination antiretroviral therapy in low-income countries: a cohort analysis from Uganda.** *Ann Intern Med* 2011, **155:**209-216.

113. Ortblad KF, Lozano R, Murray CJ: **The burden of HIV: insights from the GBD 2010.** *Aids* 2013.

114. Salomon JA, Vos T, Hogan DR, Gagnon M, Naghavi M, Mokdad A, Begum N, Shah R, Karyana M, Kosen S, et al: **Common values in assessing health outcomes from disease and injury: disability weights measurement study for the Global Burden of Disease Study 2010.** *Lancet* 2012, **380:**2129-2143.

115. Fox-Rushby JA, Hanson K: **Calculating and presenting disability adjusted life years (DALYs) in cost-effectiveness analysis.** *Health Policy Plan* 2001, **16:**326-331.

116. Murray C, Lopez A: *The Global Burden of Disease: a comprehensive assessment of mortality and disability from diseases, injuries, and risk factors in 1990 and projected to 2020.*: World Health Organisation; 1996.

117. Johnson L: **Access to antiretroviral treatment in South Africa, 2004-2011.** *South African Journal of HIV Medicine* 2012, **march:**22-27.

118. Lodi S, Phillips A, Touloumi G, Geskus R, Meyer L, Thiebaut R, Pantazis N, Amo JD, Johnson AM, Babiker A, Porter K: **Time from human immunodeficiency virus seroconversion to reaching CD4+ cell count thresholds <200, <350, and <500 Cells/mm(3): assessment of need following changes in treatment guidelines.** *Clin Infect Dis* 2011, **53:**817-825.

119. **HIV Types, Subtypes Groups and Strains** [<http://www.avert.org/hiv-types.htm>]

120. Cleary S, Blecher M, Boulle A, Dorrington R, Darkoh E, Bogopane-Zulu H: **The costs of the National Strategic Plan on HIV and AIDS & STIs 2007-2011. .** University of Cape Town, National Treasury, Broadreach Health Care and South African Parliament.; 2007

121. Meyer-Rath G: **National ART Cost Model, South Africa.** Johannesburg: Health Economics and Epidemiology Research Office, Boston University/ University of the Witwatersrand; 2011

122. Cleary S, Bouille A, McIntyre D, al. e: **Cost-effectiveness of antiretroviral treatment for HIV-positive adults in a South African township.** Cape Town: University of Cape Town, Health Systems Trust, Medecins Sans Frontieres; 2004
